# A Novel Semiconductor-Based Flow Cytometer with Enhanced Light-Scatter Sensitivity for the Analysis of Biological Nanoparticles

**DOI:** 10.1038/s41598-019-52366-4

**Published:** 2019-11-05

**Authors:** George C. Brittain, Yong Q. Chen, Edgar Martinez, Vera A. Tang, Tyler M. Renner, Marc-André Langlois, Sergei Gulnik

**Affiliations:** 1 Beckman Coulter Life Sciences, Life Science Research, Miami, FL, USA; 2 Beckman Coulter Life Sciences, Particle Characterization, Miami, FL, USA; 3 University of Ottawa Flow Cytometry and Virometry Core Facility, Ottawa, Canada; 4 Department of Biochemistry, Microbiology and Immunology, Faculty of Medicine, University of Ottawa, Ottawa, Canada; 5 uOttawa Center for Infection, Immunity and Inflammation (CI3), University of Ottawa, Ottawa, Canada

**Keywords:** Virology, High-throughput screening, Immunological techniques

## Abstract

The CytoFLEX is a novel semiconductor-based flow cytometer that utilizes avalanche photodiodes, wavelength-division multiplexing, enhanced optics, and diode lasers to maximize light capture and minimize optical and electronic noise. Due to an increasing interest in the use of extracellular vesicles (EVs) as disease biomarkers, and the growing desire to use flow cytometry for the analyses of biological nanoparticles, we assessed the light-scatter sensitivity of the CytoFLEX for small-particle detection. We found that the CytoFLEX can fully resolve 70 nm polystyrene and 98.6 nm silica beads by violet side scatter (VSSC). We further analyzed the detection limit for biological nanoparticles, including viruses and EVs, and show that the CytoFLEX can detect viruses down to 81 nm and EVs at least as small as 65 nm. Moreover, we could immunophenotype EV surface antigens, including directly in blood and plasma, demonstrating the double labeling of platelet EVs with CD61 and CD9, as well as triple labeling with CD81 for an EV subpopulation in one donor. In order to assess the refractive indices (RIs) of the viruses and EVs, we devised a new method to inversely calculate the RIs using the intensity vs. size data together with Mie-theory scatter efficiencies scaled to reference-particle measurements. Each of the viruses tested had an equivalent RI, approximately 1.47 at 405 nm, which suggests that flow cytometry can be more broadly used to easily determine virus sizes. We also found that the RIs of EVs increase as the particle diameters decrease below 150 nm, increasing from 1.37 for 200 nm EVs up to 1.61 for 65 nm EVs, expanding the lower range of EVs that can be detected by light scatter. Overall, we demonstrate that the CytoFLEX has an unprecedented level of sensitivity compared to conventional flow cytometers. Accordingly, the CytoFLEX can be of great benefit to virology and EV research, and will help to expand the use of flow cytometry for minimally invasive liquid biopsies by allowing for the direct analysis of antigen expression on biological nanoparticles within patient samples, including blood, plasma, urine and bronchoalveolar lavages.

## Introduction

Extracellular vesicles (EVs) are small, naturally occurring cell fragments that range in size between 30–1000 nm. They are generated in large numbers by living cells throughout the body, and are released as part of both normal and pathological processes. EVs are present in all bodily fluids, and their potential for use as disease biomarkers is the subject of active research in areas of major therapeutic importance, including cancer and cardiovascular disease. However, due to their small size, EVs are difficult to purify and analyze by traditional techniques^1–4^.

The most commonly used techniques for purifying EVs from blood and other bodily fluids are ultracentrifugation, size-exclusion chromatography, and PEG precipitation. Each of these are known to have biases for particular small-particle populations based on their densities, sizes, surface charges, or other properties, and each result in variable levels of residual protein and lipoprotein contamination^5,6^. Moreover, experimental characterization of the resulting samples generally consists of bulk methods, including western blots, bead-based sandwich assays, genomic assays, dynamic light scattering (DLS) and nanoparticle tracking analysis^4,7,8^. While these methods may provide insights into EV biology, they ultimately obscure individual particle characteristics and, thus, the ability to properly analyze EV populations and subpopulations. In contrast, flow cytometry is the method of choice for single-particle analyses within suspension samples, and may be uniquely suited to address the needs of the EV field^1,3^.

Flow cytometry can enable the quantitative, multiparametric characterization of EVs and other biological particles, including viruses and bacteria^9,10^. However, EVs and other biological nanoparticles typically fall within the background noise of conventional flow cytometers, which limits how useful they may be for analyzing such samples. In fact, the most sensitive conventional flow cytometers have been suggested to be unable to detect EVs smaller than roughly 300 nm in diameter^8,11,12^. Since the microvesicle size range actually extends down to 150 nm, and exosomes are said to be between 30–150 nm in diameter, this results in the common notion that only the tip of the EV iceberg can be detected by flow cytometry^1,4^.

**Figure 1:**
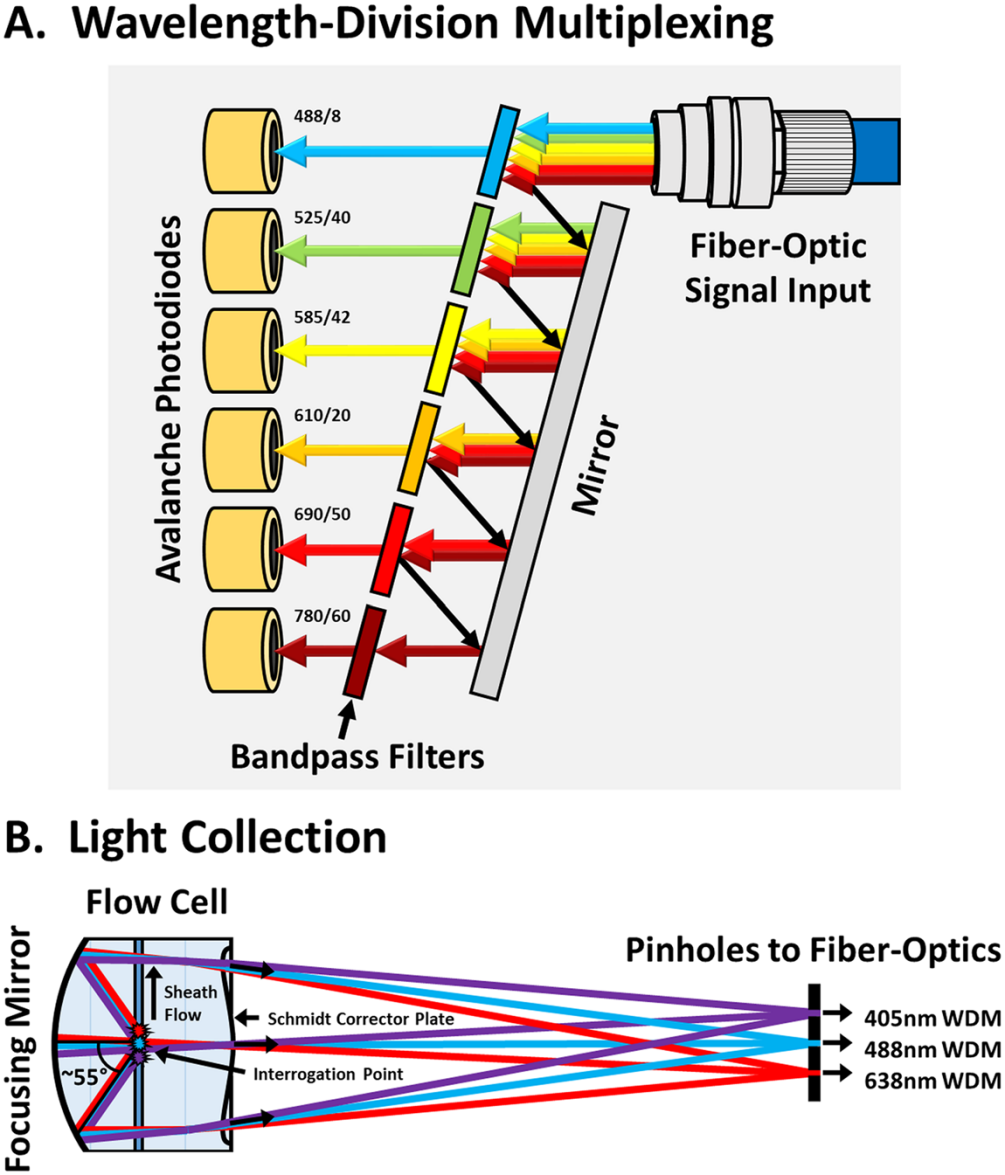
Optical Innovations in the CytoFLEX. (**A**) Wavelength-division multiplexing is a method for parsing ranges of light wavelengths, adapted from fiber-optics technology used in the telecommunications industry. Input light from the fiber-optic cable is sequentially reflected by bandpass filters until the particular wavelength range encounters a permissive filter that allows the light to pass through to its associated APD. This design minimizes the loss of light, as occurs with dichroic mirrors. (**B**) The catadioptric flow-cell design maximizes light collection. Approximately 110° of side-scatter and fluorescent light is focused by a plano-concave mirror on the back of the flow cell. The light path is then shaped by a lens designed similar to a Schmidt corrector plate, which directs light originating from the different lasers to their respective fiber-optic pinholes, while also minimizing the cross-mixing of light.

Improving upon the sensitivity of conventional flow cytometers, we have developed a semiconductor-based flow cytometer, called the CytoFLEX, which pairs silicon avalanche photodiodes (APDs) with wavelength-division multiplexing (WDM), an optimized flow-cell design, and diode lasers in order to maximize signal and minimize noise. Silicon APDs are semiconductor photodetectors that have a higher quantum efficiency and lower electronic noise than traditional photomultiplier tubes, resulting in increased light-detection sensitivity across a larger wavelength range^13–15^. The WDM design, adapted from fiber-optic technology used in the telecommunications industry, eliminates the dichroic mirrors that are traditionally used to divide light into color bands within filter trees, preventing the 20–50+% signal losses that occurs in a typical flow cytometer prior to even reaching the bandpass filters (Fig. 1A)^16^. The CytoFLEX flow cell is specially designed to maximize light capture using catadioptric optical features similar to those found within astronomical telescopes, collecting approximately 110° of side-scatter and fluorescent light, while also reducing optical noise that normally results from the cross-mixing of light from alternative laser sources (Fig. 1B)^17^. Collectively, these innovations enable the use of low-power diode lasers and small-area APDs, which further reduce electronic and thermal noise^13,18^. As a result, the performance of light-scatter detection on the CytoFLEX is so sensitive that the blue side-scatter (SSC, 488 nm) channel actually requires an attenuation filter to reduce the signal to a range useful for cells and other large particles. As an alternative, violet SSC (VSSC, 405 nm) on the CytoFLEX is unfiltered and can be used to take full advantage of the increased sensitivity for small-particle detection. Using VSSC versus SSC for small-particle detection has an added benefit that the shorter-wavelength light actually increases the amount of light scattered by small particles due to increasing the relative refractive index (RI)^19^.

In order to formally assess the light-scatter sensitivity of the CytoFLEX, we set up a series of experiments to test the detection limit for a variety of synthetic and biological nanoparticles. First, we analyzed the different modes of light-scatter detection on the CytoFLEX using a mixture of polystyrene (PS) and silica (Si) size standards, with a size range extending from 81 nm to 2 μm. We then tested a variety of small and low-scatter particles, including both synthetic beads and viruses, in order to better assess the resolution limit for VSSC on the CytoFLEX. Next, we developed a new method for converting scatter-intensity measurements to sizes and/or RIs using scaled Mie-theory curves in order to enable better characterization, and even the prediction of the sensitivity limits, for particles of different compositions. Using this method, we determined the RI of a variety of viruses and then determined the precise sizes and size ranges for the viruses. Finally, we analyzed plasma-derived EVs and compared the VSSC intensity measurements to dynamic light scattering (DLS) in order to correlate the median scatter intensities to bulk size measurements. These measurements were also used to analyze the distribution of RIs for EVs of different sizes, and we confirmed prior observations in literature suggesting that the RI of EVs increases with decreasing size.

## Results

### Assessing the different modes of light-scatter detection on the CytoFLEX

The CytoFLEX flow cytometer has 3 modes of light-scatter detection: FSC, SSC, and VSSC, which increase in sensitivity in that order. In Supplementary Fig. 1, we demonstrate the differential sensitivity of these 3 modes using a prototype mix of micro- and nanoparticles, called the CytoFLEX Standards Mix.

FSC on the CytoFLEX is not traditional small-angle light scatter, but rather a comparative signal analysis called axial light loss detection^13,20^. It functions by directly analyzing the 488 nm laser beam without an obscuration bar in order to calculate intensity differences as particles pass through the interrogation point, similar to how transit photometry is used in astrophysics to discover and characterize exoplanets in distant star systems^21^. This method has been optimized for larger particles and cell-sized events, with baseline separation for particles larger than 500 nm in diameter (Supplementary Fig. 1A). While this level of FSC sensitivity is lower than some flow cytometers, this approach was specifically designed to minimize variance and eliminate the need for routine alignment, making it more user friendly. This approach also has an added benefit that the resolution of different-sized particles using FSC is mostly independent of the RI of the particle. Indeed, while 1020 nm Si beads (RI = 1.45 at 488 nm)^22^ had a lower SSC intensity than 490 nm PS beads (RI = 1.6 at 488 nm)^23^, the FSC intensities were proportional to the volume of the particles regardless of the RI. This can be very helpful for reducing the variations in the FSC signatures of biological particles that occur due to differences in membrane integrity, such as with damaged or apoptotic cells^24,25^.

The sensitivity of SSC and VSSC on the CytoFLEX can be seen in Supplementary Fig. 1B. We found that SSC could resolve down to 214 nm Si and at least 152 nm PS particles with complete baseline resolution. The unfiltered VSSC detection was even more sensitive and could fully resolve 81 nm PS particles. Both SSC and VSSC on the CytoFLEX are true scatter-based detection methods, and the sizes of the particles that can be detected and resolved depends on their RI.

### Assessing the sensitivity of VSSC on the CytoFLEX

**Figure 2:**
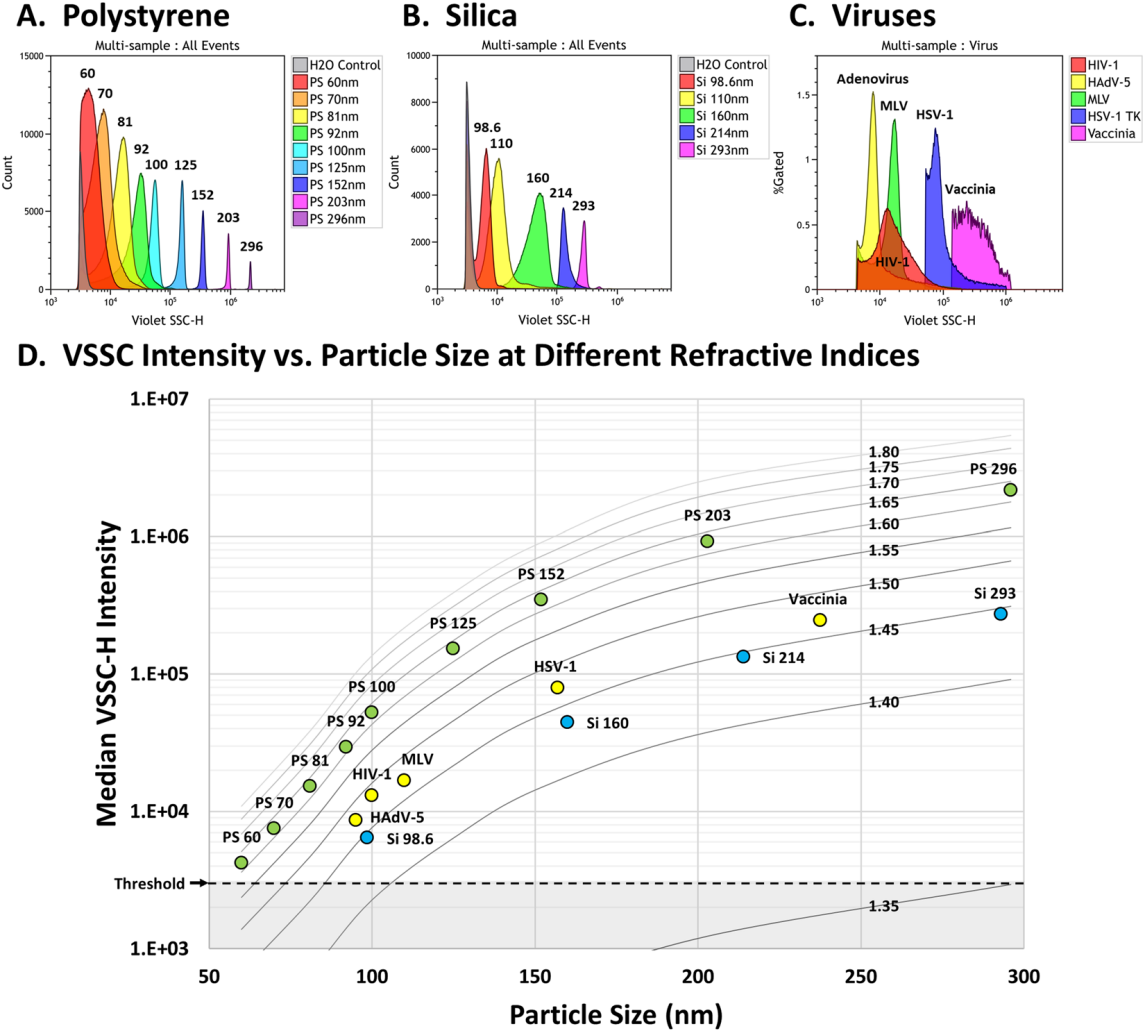
VSSC Sensitivity. (**A**) The detection of 60–296 nm PS particles by VSSC. The CytoFLEX can detect as small as 60 nm PS nanoparticles, and can resolve as small as 70 nm. (**B**) The detection of 98.6–293 nm Si particles by VSSC. 98.6 nm Si particles with a RI of 1.44 can be fully resolved by VSSC. (**C**) VSSC detection of viruses. Unlabeled 95 nm HAdV-5, 100 nm HIV-1, and 110 nm MLV can be fully resolved by light scatter on the CytoFLEX. (**D**) A contour plot prepared using Mie-theory RI curves scaled to the CytoFLEX VSSC intensities. The data from (**A–C**) were overlaid on the plot to verify the accuracy of the scaling. All samples were collected in triplicate and these data represent the population means. VSSC gain = 400; VSSC-H threshold = 3000. The VSSC-H threshold for HSV-1 and Vaccinia was 40 K and 100 K, respectively.

In order to better assess the baseline sensitivity of VSSC on the CytoFLEX, we next tested a variety of synthetic and biological nanoparticles with different RIs (Fig. 2). For PS nanoparticles, we found that the CytoFLEX could detect PS beads as small as 60 nm, though these beads did not achieve baseline separation (Fig. 2A). PS beads as small as 70 nm were resolved from noise. For Si nanoparticles, the CytoFLEX could fully resolve 98.6 nm Si beads with a RI of 1.44 (Fig. 2B). Analyses of viral particles revealed that the CytoFLEX can fully resolve Human Adenovirus-5 (HAdV-5), Human Immunodeficiency Virus-1 (HIV-1), and Murine Leukemia Virus (MLV) (Fig. 2C). HAdV-5 is a non-enveloped DNA virus that is approximately 95 nm in diameter^26,27^. HIV-1 is an enveloped retrovirus that has a peak diameter of approximately 100 nm^28,29^, but a broad size range resulting in a heavily skewed mean. MLV is another enveloped retrovirus with a diameter of approximately 110 nm^9,30,31^. Herpes Simplex Virus-1 (HSV-1) is a larger DNA virus, with a diameter range between 125 to 250 nm, and a peak tegument distribution between 138 to 176 nm, averaging 157 nm^32,33^. Vaccinia Virus (VV) is a large, ellipsoidal DNA virus, with highly variable size characteristics in literature. In order to assign an approximate spherical diameter to VV for Mie-theory analysis, we calculated the ellipsoidal volumes for VV with size characteristics from 5 references, estimated the diameters for spheres of equivalent volumes, and then determined the average spherical diameter to be 237.5 nm (Supplementary Table 1)^34–38^. The average measurements for each particle are listed in Supplementary Table 2, and more detailed information is in Supplementary Fig. 2.

### A method for converting scatter intensity to size or RI

The quantity of light scatter produced by particles depends directly on their RI, which complicates the ability to determine the size of particles based on reference standards with a different RI. Synthetic reference standards are still useful, though, because they have well-defined characteristics that remain constant regardless of the instrument or detection method. In order to help improve the characterization of small particles with different compositions by flow cytometry, we devised a simple method for approximating the size or RI of particles based on their scatter intensity and one or the other parameter. Standard approaches to determining particle characteristics based on light scatter and inverse Mie-theory calculations require a variety of instrument variables that may not be known or readily available^39,40^. Because all of these variables are contained within the real scatter intensities produced by the instrument, we hypothesized that we could actually use reference standards to scale Mie-theory curves to the scatter intensities expected for the flow cytometer. We calculated the expected scatter intensities by creating ratio equivalencies of the real intensities for the reference particles divided by their theoretical scatter efficiencies, and then solving for the intensities of particles of the same sizes but different RIs. Since the conversion of scatter intensities between RIs involves a simple ratio of real-versus-calculated intensities, any unknown variables that contribute to the signal cancel out as constants.

A proof of concept for our conversion method is demonstrated in Fig. 2D for the VSSC detection of a variety of nanoparticles on the CytoFLEX. We first measured a set of reference particles with known characteristics, including 60, 81, 100, 125, 152, 203 and 296 nm NIST-traceable PS beads (Supplementary Fig. 3). The RI of PS was calculated to be 1.627 at 405 nm^41^, while the RI for water was calculated to be 1.3388^42^. The average scatter intensities and theoretical scatter efficiencies for each particle are listed in Supplementary Fig. 4A. Next, we prepared a matrix of theoretical scatter efficiencies for the particle sizes with RIs between 1.35 and 1.80 (Supplementary Fig. 4B), and then calculated the approximate VSSC intensities for each particle at the different RIs (Supplementary Fig. 4C). Finally, the matrix of calculated VSSC intensities was plotted versus size, with the different curves representing contours of RI equivalencies (Fig. 2D). The accuracy of our method was confirmed by plotting the nanoparticles from Fig. 2A–C into the contour plot: the PS and Si particles fall right into their expected RI ranges, around 1.63 and 1.44, respectively^43^. Interestingly, the RIs for all of the viruses were found to be approximately 1.47 based on the contours. While the RIs for these viruses are not available in literature, and viral characteristics vary, this value is within the middle range for viruses that have been characterized by alternative methods^44^. The exact sizes and RIs can be more precisely calculated by fitting equations to the curves and solving for the unknown variable. In this case, using more precise calculations based on the virus sizes from literature, we determined the RIs at 405 nm to be: 1.474 for HAdV-5, 1.484 for HIV-1, 1.469 for MLV, 1.470 for HSV-1, and 1.466 for VV (Supplementary Figs 5 and 6), averaging 1.473. A common refractive index between viruses would appear to suggest that it correlates to their protein content because the differences in virus size largely represent different quantities of protein. The minor differences in the calculated RIs may be due in part to the imprecision of using approximate sizes from literature. The lower detection limit for additional particles with a RI of 1.47 would be approximately 81 nm at the threshold and 86 nm at the level of 60 nm PS beads (Supplementary Fig. 7).

### Characterizing virus sizes by flow cytometry

**Figure 3:**
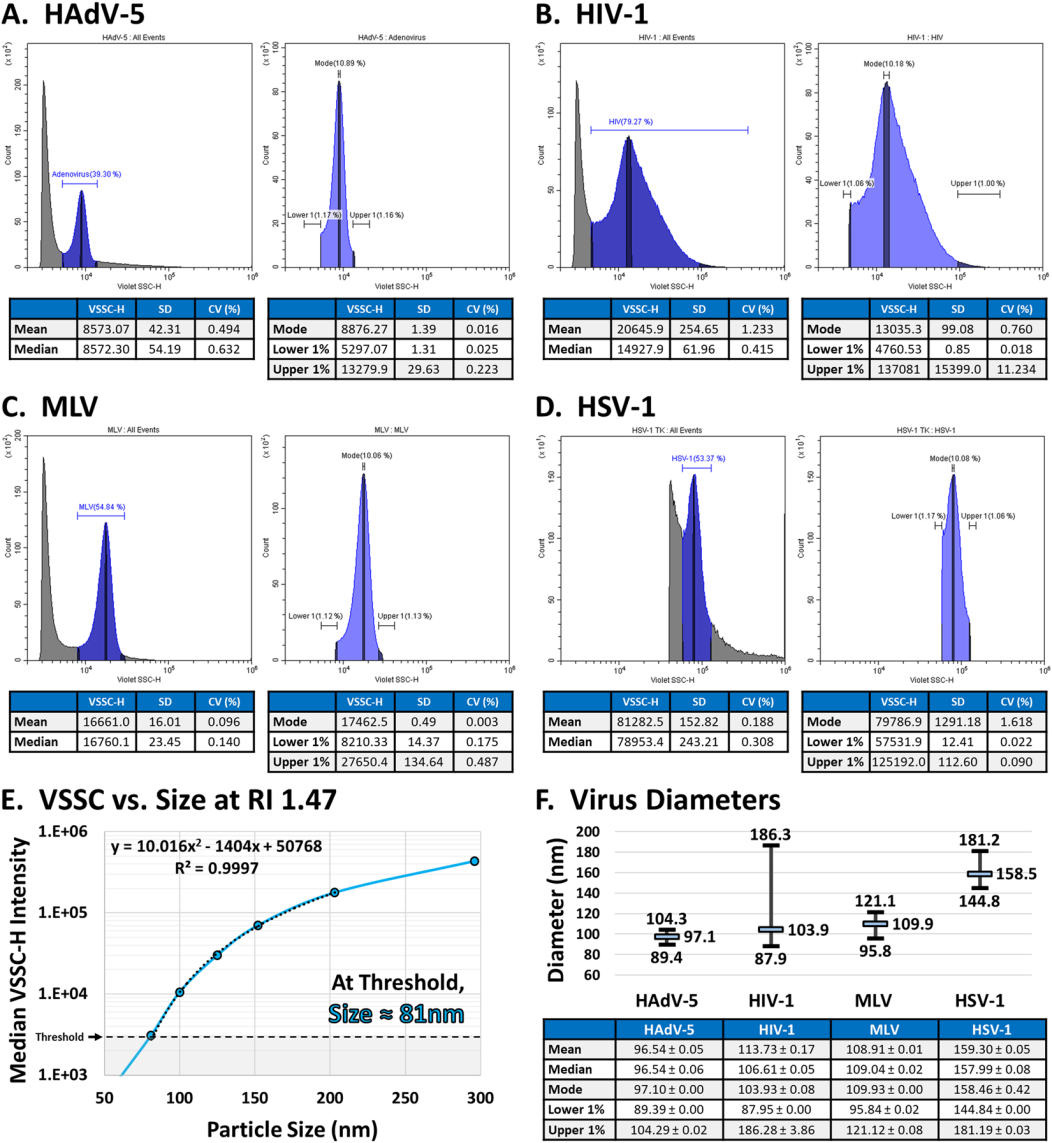
Sizing Viruses by Flow Cytometry. (**A–D**) VSSC-H population statistics for (**A**) HAdV-5, (**B**) HIV-1, (**C**) MLV, and (**D**) HSV-1. Each virus was read in triplicate and the data represent the average. (**E**) Fitting a VSSC-H Intensity vs. Size curve for RI 1.47 at 405 nm in order to calculate virus sizes. (**F**) The calculated size characteristics for each virus in (**A–D**). In the chart above, the calculated peak diameter is displayed along with the size range. The mean and median sizes are also included in the table below, ±the SEM.

Since each virus appears to have a common refractive index, and because virus sizes are variable and sometimes imprecise in literature, we next set out to calculate the size characteristics for each virus tested based on their VSSC measurements. The mean, median, and mode VSSC measurements, as well as upper and lower limits can be found in Fig. 3: HAdV-5 (A), HIV-1 (B), MLV (C), and HSV-1 (D). VV was excluded simply due to its asymmetrical shape. The equation fitting the estimated VSSC intensities for 81–200 nm particles at a 1.47 RI can be found in Fig. 3E. Using this equation, the sizes of the viruses were calculated in Fig. 3F. Each of the calculated diameters and size ranges conforms well to literature, and may actually have improved accuracy due to sampling hundreds of thousands of intact events rather than a few-hundred hand-selected events from micrographs of highly-processed samples.

### Analyzing plasma-derived EVs on the CytoFLEX

In order to determine the detection limit for plasma-derived EVs on the CytoFLEX, we combined a series of sample-preparation approaches to narrow down the size range of the plasma-EV sample fractions. First, we prepared platelet-poor plasma (PPP) by centrifugation. Then, we filtered out any residual large particles using a 0.2 μm filter. Finally, we performed size-exclusion chromatography (SEC), using Izon columns with a 70 nm pore size, to reduce proteins and lipoproteins. Using this approach, we narrowed the size range down to roughly 70–200 nm for comparison between DLS and flow cytometry.

**Figure 4:**
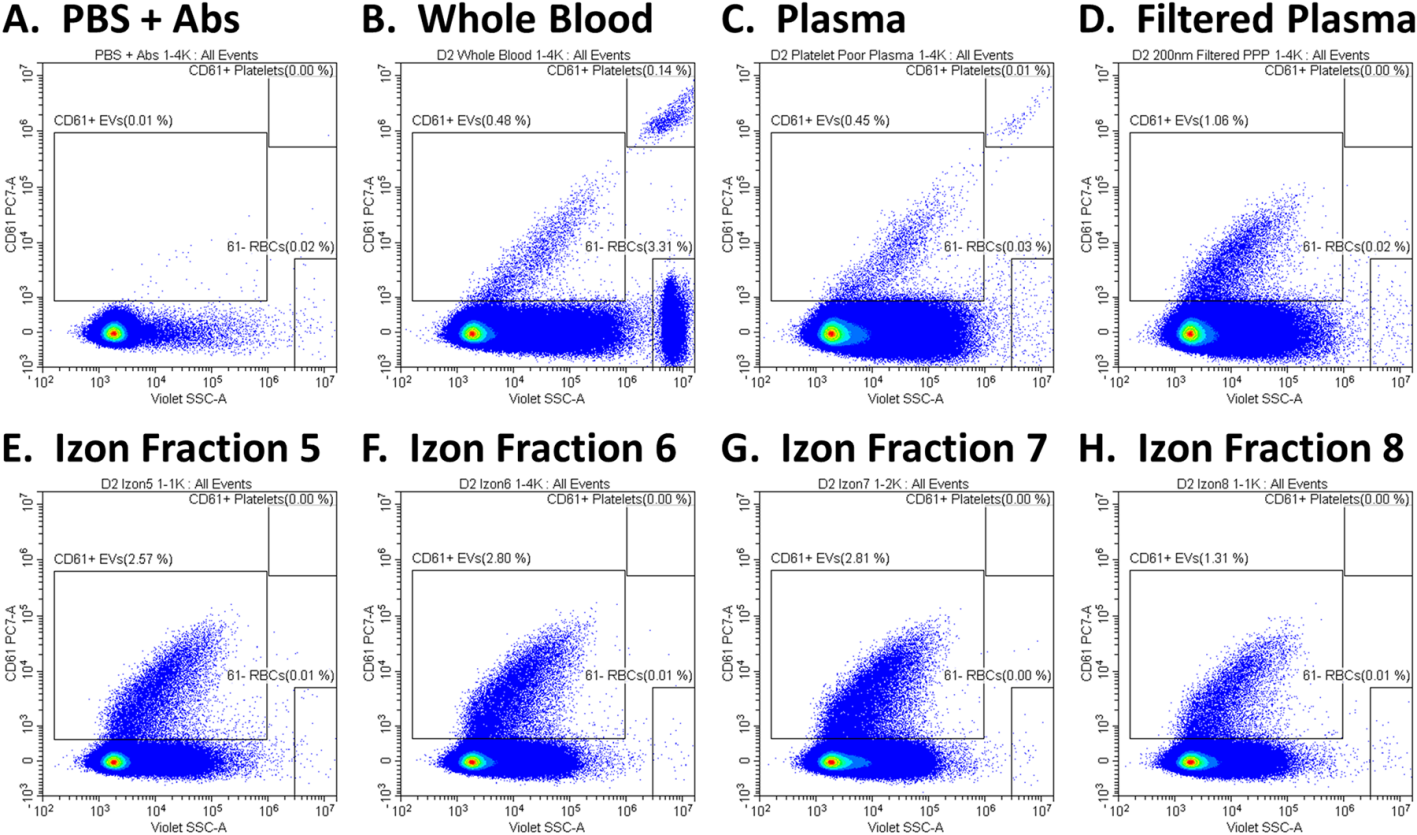
Preparation of Plasma EVs. (**A**) PBS + antibody cocktail as a control to demonstrate the absence of fluorescent-antibody aggregates. (**B**) CD61^+^ EVs and platelets in whole blood. (**C**) CD61^+^ EVs in PPP, with RBCs eliminated and platelets depleted. (**D**) CD61^+^ EVs in PPP filtered through a 0.2 μm filter, with platelets eliminated. (**E–H**) CD61^+^ EVs in the 5^th^ through 8^th^ 200 μL fraction from 0.2 μm-filtered PPP passed through an Izon qEV column. Serial dilutions were performed and all samples were acquired in triplicate. VSSC gain = 400; VSSC-H threshold = 3000.

**Figure 5:**
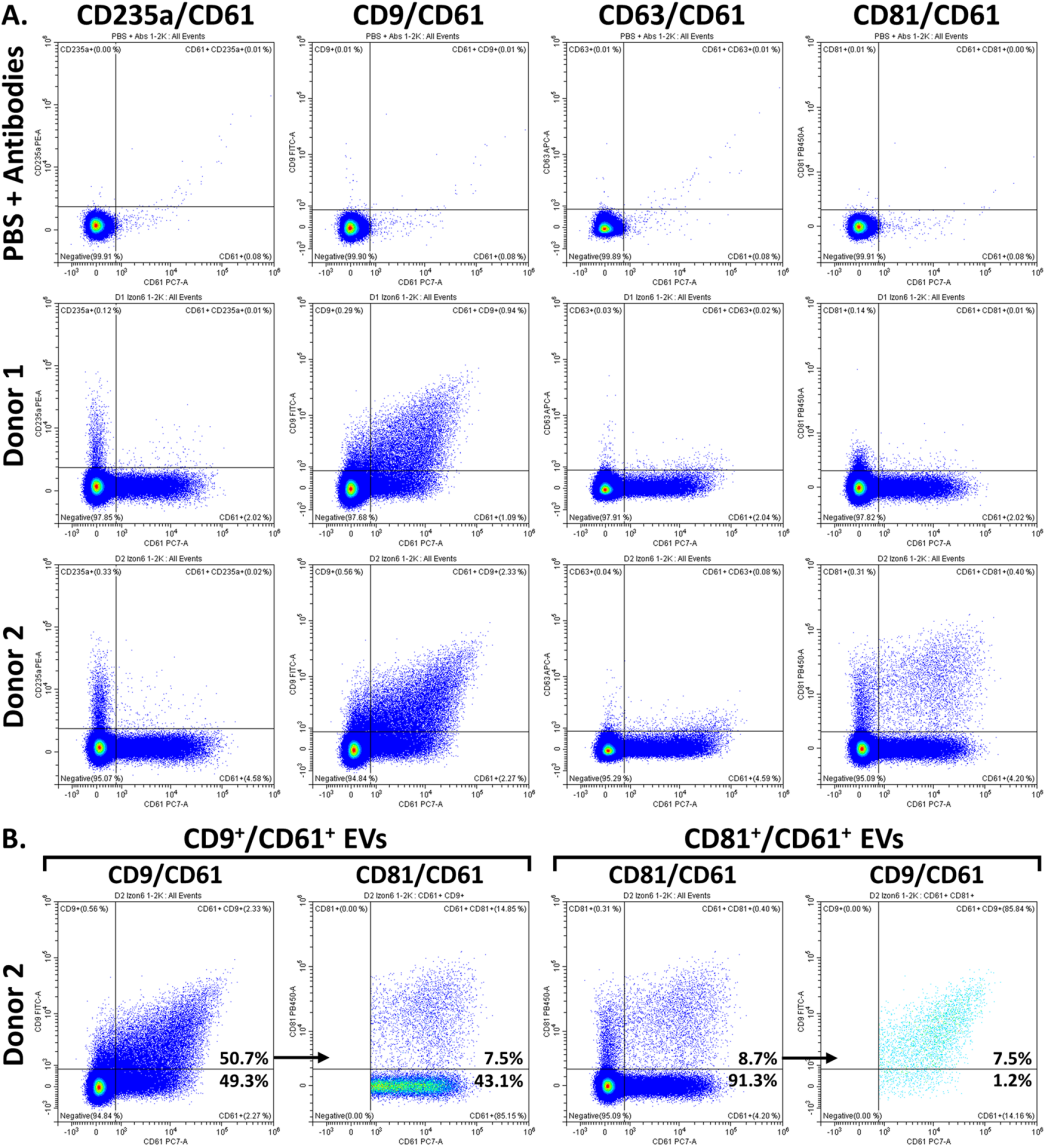
Tetraspanin Expression on the CD61^+^ plasma EVs. (**A**) CD61^+^ plasma EVs from human donors were tested for their expression of CD9, CD63 and CD81. The CD61^+^ EVs were around 50% CD9^+^, while CD63 and CD81 expression was mostly absent. 8.7% of the CD61^+^ EVs from Donor 2 were CD81^+^. The PBS + antibody control demonstrates the lack of fluorescent-antibody aggregates. (**B**) Further analysis of the CD81^+^ EVs revealed that these were predominantly CD9, CD81 and CD61 triple positive. This is reciprocally demonstrated by analyzing the CD81 expression on CD9^+^ CD61^+^ EVs, and the CD9 expression on CD81^+^ CD61^+^ EVS. In total, 7.5% of the CD61 EVs from Donor 2 were triple positive. The percentages in bold are in reference to the overall CD61^+^ EV population. These samples were serially diluted and acquired in triplicate. VSSC gain = 400; VSSC-H threshold = 3000.

The effectiveness of this approach can be seen in Fig. 4. CD61^+^ platelets and platelet EVs in whole blood are shown in Fig. 4B^45^. The RBCs were then eliminated, and the platelets reduced, by centrifugation to prepare PPP (Fig. 4C). By filtering the PPP through a 0.2 μm filter, the residual platelets and larger microvesicles were eliminated (Fig. 4D). The filtered PPP samples were then passed through Izon columns, and fractions 5 to 8 were collected as the purified-EV sample fractions. In each of these samples, the amount of background protein and lipoprotein appears reduced, and the relative % of CD61^+^ EVs increased between 2- to 5-fold over plasma (Fig. 4E–H). Titrations of the Izon fractions, together with the population statistics, can be found in Supplementary Fig. 8. The CD61^+^ EVs prepared using this method were roughly 50% CD9 double positive, while they were predominantly CD63 and CD81 negative (Fig. 5A and Supplementary Fig. 9). Approximately 8.7% of the CD61^+^ EVs from Donor 2 were CD81 double positive, including 7.5% that were CD9, CD81 and CD61 triple positive (Fig. 5B). However, the majority of donors appear more similar to Donors 1, 3 and 4. The RBC marker, CD235a, was included as a negative control for platelet EVs, while the PE conjugate was specifically used to control for antibody or protein aggregation in the case of multi-labeled events, since PE is particularly prone to aggregate: the CD61^+^ EVs were all negative for CD235a-PE, which supports the specificity of the double and triple labeling.

**Figure 6:**
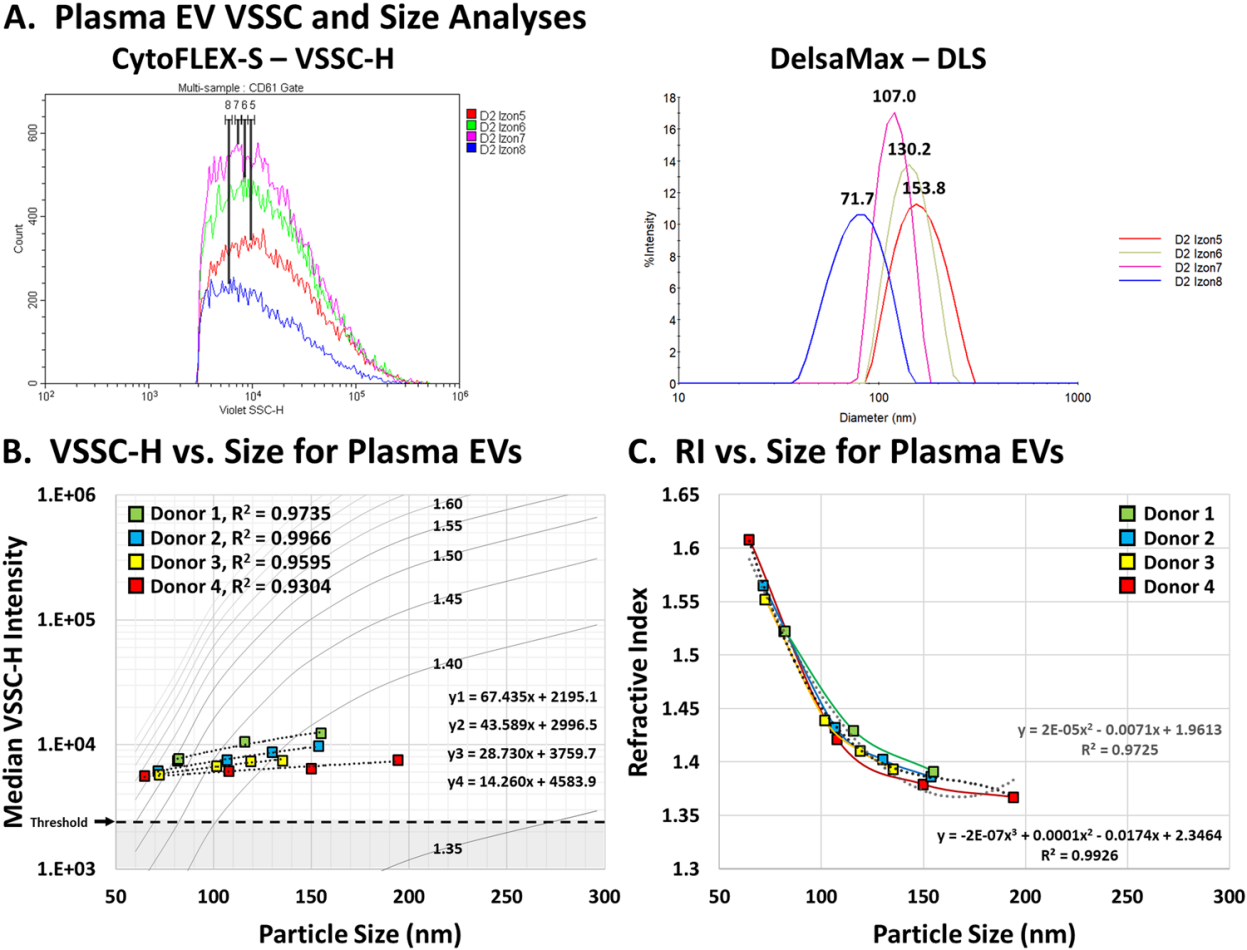
Analysis of the VSSC Sensitivity for EV Detection. (**A**) Analysis of the purified EV fractions by VSSC intensity and DLS sizing. The overlay plots demonstrate the differential VSSC intensities and DLS sizes for representative measurements of the EV fractions from Donor 2. (**B**) Overlay of the average VSSC intensity vs. DLS size measurements for each EV fraction on the scaled Mie-theory RI curves prepared in Fig. 2. The sample distributions are almost perfectly linear, with R^2^ values between 0.930 and 0.997 relative to linear trendlines. (**C**) The RIs of the EV fractions increase progressively as their sizes decrease. All samples were serially diluted and the VSSC-H intensity measurements were acquired in triplicate or quadruplicate, while 100 DLS measurements were acquired per sample. VSSC gain = 400; VSSC-H threshold = 3000.

A portion of each sample from Fig. 4E–H was also analyzed by DLS in order to characterize the sizes of each sample fraction. Figure 6A shows representative overlays for both the flow-cytometry and DLS data from the EV fractions. The average median VSSC and size readings can be found in Supplementary Figs 8 and 10. The median sizes for the EV fractions were determined to be between 65–194 nm, confirming the expected bounds for the purification approach. These data were plotted on the scaled Mie-theory RI contour plot prepared in Fig. 2, and interestingly had a perfect linear correlation for each donor within this size range, with R^2^ values between 0.930–0.997 (Fig. 6B). If this linear trend for the EV fractions extends lower, then EV detection on the CytoFLEX should extend down to at least 32.6 nm at the level of 60 nm PS beads, and 12 nm at the threshold level, depending on donor-specific differences in the EV RIs. However, it must be noted that these EV characteristics are average population statistics composed of a distribution of many individual characteristics. We actually calculated the RIs for each EV fraction and found them to range between 1.608 at 64.8 nm and 1.367 at 194.3 nm (Fig. 6C). Further details on the RI calculations for each donor can be found in Supplementary Figs 11–14. This increase in the RI of EVs at smaller sizes is expected due to the increasing ratio of macromolecules to H_2_O content^43^, and comports well with literature^46^.

## Discussion

We demonstrated that the CytoFLEX flow cytometer can effectively detect and resolve nanoparticles and plasma-derived EVs well in to the exosome range: 30–150 nm. By VSSC, we were able to fully resolve 70 nm PS beads and 98.6 nm Si beads, as well as HAdV-5, HIV-1 and MLV. To our knowledge, no other flow cytometer has ever been demonstrated to resolve adenoviruses and HIV, or PS and Si beads within this size range, by light scatter. MLV detection has been demonstrated on another flow cytometer^9^, but the CytoFLEX has much improved resolution and consistency by comparison. Moreover, we found that the CytoFLEX can detect EVs at least as small as 65 nm, triggering with VSSC and immunophenotyping to identify the smallest EV populations. This level of sensitivity can greatly benefit exosome and EV research, as most flow cytometers have been suggested to be unable to detect EVs smaller than 300 nm in diameter^11^. In fact, while our results demonstrate that the CytoFLEX is more sensitive than even dedicated microparticle analyzers^45,47,48^, it is indeed a full-fledged flow cytometer with a complete dynamic range for cells and other large particles.

The single biggest innovation that enables such dramatic performance improvement on the CytoFLEX is the use of small-area APDs for signal detection. Small-area APDs have a higher quantum yield, increased linear dynamic range, and minimal dark-current electronic noise^13–15^. The CytoFLEX was the first-in-class flow cytometer to replace traditional PMTs with APDs, and the performance and sensitivity improvements that have been demonstrated ever since have paved the way for others to follow suit. Due to the enhanced sensitivity provided by the APDs, the rest of the optical bench was built around enhancing performance on the lower end of the signal spectrum, rather than brute-force modifications to increase signals, such as with the use of higher-powered lasers. The design of the WDM module on the CytoFLEX, another first-in-class, is an innovative adaptation of fiber-optic technology from the telecommunications industry, allowing for the light signals on the CytoFLEX to be directed to their respective APDs without passing through any light filters or mirrors prior to reaching their final destination (Fig. 1A)^16^. This design allows for maximal signal collection. Conversely, the filter trees in traditional flow cytometers lose 20% or more light per dichroic mirror used to divide the light into color bands, which can result in >50% signal losses for some channels. The design of the flow cell similar to a miniature catadioptric telescope is another first-in-class innovation to maximize light collection, enabling the collection of 110° of side-scatter and fluorescent light (Fig. 1B)^17^. Traditional flow cytometers generally collect only 30–60° of side-scatter and fluorescent light, typically using focusing lenses. Finally, the enhanced signal collection and sensitivity improvements provided by the combined use of small-area APDs, WDMs and the optimized flow-cell design allows for the use of low-power diode lasers, which results in less electronic and thermal noise^18^. The combined result is a novel, 21^st^-century, semiconductor-based flow cytometer with robust performance, extreme reliability without the need for constant maintenance and alignment, and improved light-scatter and fluorescence sensitivity, largely enhancing the signal detection on the lower end of a 7-decade dynamic range.

Interestingly, there has been a recent trend where researchers and companies are shifting toward utilizing VSSC for small-particle detection on a variety of instruments. This is presumably due to an increased awareness of the enhanced VSSC sensitivity found on the CytoFLEX, together with knowledge of the Sellmeier observation that the refractive index of a material depends on the wavelength of incident light, particularly that using lower-wavelength light can increase the light scatter of small particles^19^. However, VSSC alone may not provide much benefit toward increasing the sensitivity of such instruments beyond a marginal enhancement in light refraction, which can be easily offset by differences in laser power and other factors. The biggest factor contributing to the enhanced sensitivity of VSSC on the CytoFLEX is simply that the VSSC signal is not attenuated like SSC. The enhanced sensitivity of the CytoFLEX vs. other instruments, even when using diode lasers with less than half of the power output of traditional lasers, is again due to the enhanced optics collecting approximately 110° of orthogonally scattered light, the WDMs minimizing signal losses, and the small-area APDs having a high quantum yield with minimal electronic noise. Some of these factors may have no substantial benefit on their own, but they combine to great effect.

The approach that we developed for converting light-scatter intensity measurements to approximate sizes or RIs is relatively simple and should work with any light-scatter parameter. While forward Mie-theory calculations to predict the light-scatter efficiency of particles can be complex, they are much easier than the inverse calculations needed to determine particle characteristics based on light scatter^49,50^. Indeed, solving Mie-theory equations to determine specific particle characteristics typically requires a variety of complex variables pertaining to the internal engineering of the flow cytometer, which may not be readily available^39,40,45,51,52^. Moreover, Mie-theory solutions have been said to provide a lot more information than necessary, such that relying too heavily on theoretical calculations rather than real measurements can complicate the interpretation of results; e.g., predicting ripples in scatter profiles that may be due to imaginary or radiative components, but are not observed in actual measurements^49,53^. With our approach, scaling the forward Mie-theory calculations to actual reference-particle intensities, we addressed the inverse source problem by simply canceling out any unknown instrument variables as constants. Using this approach, we were able to effectively characterize the sensitivity of the CytoFLEX for biological and synthetic particles of different compositions, and even found that flow cytometry can be an effective tool for rapidly and accurately sizing viruses based on their apparent common RI: approximately 1.47 at 405 nm. Further testing will be needed to verify this result since it is certainly possible that there are deviations, particularly outside of the size range tested or with different experimental conditions.

Our current research on the CytoFLEX optics involves further improving the scatter and fluorescence sensitivity, with the goal of developing an analyzer that can provide complete baseline resolution for the smallest biological nanoparticles, including exosomes and small viruses. However, with thoughtful sample preparation and proper instrument operation, we have found that the current state-of-the-art CytoFLEX can already enable the detection and analysis of a variety of nanoscale viruses and EVs at the single-particle level, including EVs directly in whole blood and plasma. This enhanced functionality is perhaps uniquely capable of helping scientists and clinicians to achieve their goal of developing EV-based liquid biopsies for hard-to-detect diseases, such as tissue-resident cancers, minimal residual disease, and even neurological disorders. EV-based companion diagnostics also promise to provide for earlier diagnoses of diseases, such as heart attacks and strokes, better stratification and prediction of therapeutic outcomes, and improved analyses of disease progression, any one of which would revolutionize the future of personalized medicine^1,2,4^.

## Methods

### Blood

Fresh blood was collected onsite daily by Blood Services using K3-EDTA Vacutainers (Becton Dickinson, Franklin Lakes, NJ). All samples were obtained from normal adult human donors under signed informed consent, as per Western Institutional Review Board-reviewed and -approved protocol. All methods were performed in accordance with the relevant guidelines and regulations.

### Reagents

The CytoFLEX Standards Mix is a prototype bead mix from Beckman Coulter (Brea, CA), consisting of 81, 100, 152, and 296 nm NIST-traceable polystyrene (PS) beads, 214, 1020 and 2000 nm NIST-traceable silica (Si) beads, and 100, 196 and 490 nm green-fluorescent PS beads. The 60 (3060A), 70 (3070A), 81 (3080A), 92 (3090A), 100 (3100A), 125 (3125A), 152 (3150A), 203 (3200A) and 296 nm (3300A) NIST-traceable PS beads were from ThermoFisher Scientific (Waltham, MA). The 68.6 (NS-0070A) and 98.6 nm (NS-0100A) NIST-traceable Nanosilica beads were from MSP Corporation (Shoreview, MN). The 110 nm Si beads were from Sigma Aldrich (803308, St. Louis, MO). The 160 (SS02000) and 293 nm (SS02001) Si beads were from Bangs Laboratories (Fishers, IN). The 214 nm Si beads were obtained from Corpuscular (140140-10, Cold Spring, NY). Live HAdV-5 was purchased from Vector Biolabs (1060, Malvern, PA). Formalin-inactivated HIV-1 (HV-H-Zero) and MLV (MV-M-Zero) were obtained from ViroFlow Technologies, Inc. (Ottawa, Canada). The HSV-1 and VV were custom viral preparations prepared by freeze/thaw cell fracturing^10^, also from ViroFlow Technologies, Inc. CD9-FITC (IM1775U), CD235a-PE (IM2211U), CD61-PC7 (IM3761), and CD81-PB (B19717) were from Beckman Coulter (Brea, CA). CD63-APC was from BioLegend (353008, San Diego, CA). Izon qEVsingle/70 nm SEC columns were purchased from Izon Science LTD (SP2, Oxford, United Kingdom).

### Nanoparticle preparation

Nanoparticles were diluted from their stock concentrations into HPLC water (WX0008-1, MilliporeSigma, Burlington, MA) before running on the flow cytometer. In order to find the appropriate working concentrations, initial 1:100 or 1:1000 concentrations were prepared, and these were then serial diluted at a 1:2 ratio until swarming was minimized or eliminated and the signal-to-noise ratios were optimal.

### Plasma EV sample preparation

Plasma was prepared from fresh human blood by a combination of centrifugation and filtration. Whole blood was aliquotted into 12 × 75 mm tubes and then PPP was prepared using a 2-step process. First, the blood was centrifuged for 5 min at 160 × g in an Allegra 6 R Centrifuge (366816, Beckman Coulter, Brea, CA), and the upper supernatant was collected as PPP, careful to avoid collection of platelet-rich plasma near the WBC layer^54^. Rather than subjecting the PPP to additional centrifugation steps that can also eliminate EVs, the residual platelets and larger particles were then eliminated by filtering the collected PPP through a 0.2 μm Acrodisc syringe filter #4612 (Pall Corporation, Port Washington, NY). The filtered PPP was then passed through Izon qEVsingle SEC columns with a 70 nm pore cutoff in order to eliminate the abundance of proteins and lipoproteins present below the size cutoff for the columns, while further narrowing the size distributions in the different column fractions. SEC was performed according to the manufacturer’s instructions. Briefly, the columns were first flushed with 1x PBS (1408, Sigma-Aldrich, St. Louis, MO), 150–200 μL of PPP was then applied to the column, and this was followed by the addition of PBS to elute the sample fractions. The initial 800 μL of column eluate was voided, while further 200 μL fractions were collected for analysis by both DLS and flow cytometry.

### Antibody labeling

Immunophenotyping was performed on the plasma-EV samples to identify the populations of interest. To label with antibodies, the antibodies were first mixed in their appropriate concentrations in a master mix. The optimal concentrations were empirically determined for each antibody. 50 μL of plasma or EV samples were aliquotted into different 12 × 75 mm tubes, and an aliquot of the antibody mixture was added to each sample. The samples were then allowed to incubate for at least 1-hour in the dark at RT. After labeling, the samples were diluted into flow-cytometry resuspension buffer (1x PBS + 0.2% PFA) and aliquotted into a 96-well plate for flow-cytometric analyses. Depending on the preparation method and particular donor, the appropriate dilution for the plasma-EV samples prior to analysis was typically within the range of 1:1 K to 1:4 K relative to the initial plasma when running at a medium sample rate (30 μL/min). In order to accommodate for donor-to-donor differences, all samples were serially diluted and acquired at a variety of dilutions between 1:250 and 1:4 K.

### Flow cytometry

All experiments in this study were performed using a 13-color, 4-laser CytoFLEX S N-V-B-R Flow Cytometer, equipped with 375 nm, 405 nm, 488 nm and 638 nm lasers (B78557, Beckman Coulter, Brea, CA) (research use only; not for diagnostics), and operated using CytExpert Software v1.2 (Beckman Coulter, Brea, CA). For small-particle analysis, the configuration was modified for VSSC detection. Briefly, the 405/10 VSSC filter was moved to the V450 channel in the WDM, while the V450 and V525 channels were each shifted (eliminating the V610 channel), and the detector configuration was modified in the CytExpert software to assign the VSSC channel within the WDM. The Event-Rate Setting was set to High prior to initiating analyses, tightening the pulse window and thus reducing the background for small-particle analyses. Finally, the trigger channel was set to VSSC-Height and the threshold level was manually set as appropriate for the small particles. The particular threshold setting for different instruments directly correlates to the laser power and VSSC gain, and depends on how much optical noise the particular user prefers in the background, but it is usually in the range of 10x the gain setting. The optimal threshold setting for the CytoFLEX S N-V-B-R was determined empirically using 81 nm PS nanoparticles at their optimal dilution. Most experiments in this study were conducted using a VSSC gain of 400 with a VSSC-H threshold of 3000.

Prior to running the small-particle experiments, the sample probe was cleaned to reduce any debris. This step is particularly important for small-particle experimentation because any debris in the sample lines will increase the background noise and potentially swarm with the population of interest. Most noise of this nature resides in the scatter range below 100 nm PS beads, so this may not be a notable issue for less sensitive flow cytometers. For semi-automatic acquisition mode, cleansing was performed using a panel of bleach, FlowClean Cleaning Agent (A64669, Beckman Coulter, Brea, CA) and then two tubes of clean water to flush out the remaining detergent and debris, at the max rate for 1–2 minutes each. For plate-loader mode, this was performed using 2x alternating wells of FlowClean and water, followed by two additional wells of water to finish flushing any residual detergent and debris prior to the first sample. These steps were repeated if necessary.

### Dynamic light scatter

DLS experiments were performed using a DelsaMax Pro Analyzer (B29164, Beckman Coulter, Brea, CA) according to the manufacturer’s instructions. Following SEC, 100 μL of sample was aliquotted into a cuvette, and each sample was read 10x with 10 acquisitions per read (100 acquisitions in total). If necessary, the samples were diluted as much as 1:20 in 1x PBS. All acquisitions were performed for 2-seconds each at 25 °C, with the peak radius between 0.5 to 10,000 nm and the autocorrelation function set to between 2.0 to 200,000 μs. The median diameter for each sample was calculated using cumulants analyses on the % Intensity measurements from Brownian motion using the Rayleigh Spheres model. Outliers and skewing were minimized for the DLS readings by preparing the samples with narrow size distributions, as previously described. The DelsaMax Pro was standardized using 68.6 (NIST), 98.6 (NIST), 160 and 214 nm Si particles (Supplementary Fig. 15).

### Data analyses

All flow cytometry data were analyzed using CytExpert v2.3 and Kaluza v1.5 (Beckman Coulter, Brea, CA). First, the compensation matrix was fine-tuned for the samples, if applicable. Next, the population gates were adjusted for each sample, a statistics table was prepared, and the population data were then exported to prepare charts and graphs using Excel 2013 (Microsoft, Redmond, WA). DLS data were analyzed using DelsaMax Software v1.6.1.17 (Beckman Coulter, Brea, CA).

### Statistical analyses

All statistical analyses were performed in Excel 2013. Each sample was read in triplicate or more, and the population means were calculated by averaging the median or mean intensity measurements from each individual data point. The standard deviation for each sample was calculated using the STDEV function, which calculates ${\rm{SD}}=\sqrt{\frac{{\sum }_{i=1}^{N}{({x}_{i}-\bar{x})}^{2}}{N-1}}$. The coefficients of variation (CVs) as a percentage, ${\rm{C}}{\rm{V}}({\rm{ \% }})=(\frac{SD}{Mean})\times 100{\rm{ \% }}$. The standard error margin $({\rm{SEM}})=\frac{SD}{\sqrt{N}}$.

### Mie-theory conversions

The scatter efficiencies were calculated using the Fortran-based, Wiscombe Mie-theory code, MIEV0, available online from the Oregon Medical Laser Center (omlc.org/software/mie/). The theoretical scatter efficiencies at 405 nm were calculated for a variety of particles sizes (equivalent to the PS standards), and at multiple RIs between 1.35 and 1.80 (in 0.05 increments). The RI for PS was calculated to be 1.627 at 405 nm^41^, and the RI for water was calculated to be 1.3388 at 405 nm^42^. After the VSSC-H intensities were measured for the PS reference particles, the matrix of Mie-theory scatter efficiencies was converted to scaled VSSC intensities by calculating equivalency ratios between the reference standards and the values for equivalently size particles at the different RIs. The theoretical intensities were calculated by solving the equivalency ratios for the unknown variable, as follows:

$$Theoretical\,Intensity=(\frac{Actual\,VSSC\,Intensity\,}{Reference\,Scatter\,Efficiency\,})\times Theoretical\,Scatter\,Efficency.$$

This scaling approach does not require an exhaustive number of reference beads, but the more points that are included within the targeted size range, the more accurate the contours and calculations will be.

Calculations of either the RIs or the sizes of the different particles can be accomplished in a similar manner. If multidimensional analysis software is not available, the unknown values can be calculated manually by focusing in on one dimension at a time. In order to calculate the RI of particles based on their intensity measurement and size, an equation should first be best fit to the reference curve. This equation can then be solved for the approximate scatter intensity of a reference particle at the size of interest. Next, a matrix of Mie-theory scatter efficiencies is prepared for different RIs using the size of interest, and this matrix is converted to scaled instrument intensities as above, using the approximate scatter intensity previously calculated as the reference intensity. The best-fit equation for this intensity curve can be solved to determine the RI of the particle at the experimentally measured intensity. Since determining small differences in the decimal places of a RI vs. non-linear differences in scatter intensities many orders of magnitude greater can be difficult and inaccurate using polynomial equations, a simpler alternative is to focus in on the estimated RI range based on the main contour plot, and then use smaller RI increments, such as 0.01, focused within the range of interest. The 2 RI increments on either side of the experimentally measured intensity can be identified, and a linear equation between these 2 points can be easily solved to closely approximate the RI of the sample. This method is similar to solving for the tangent of a curve in calculus, where the tighter the interval gets, the more the curve approaches a straight line that provides for a reasonably accurate localized slope. Curves demonstrating such a linear tangential fit were prepared by overlaying 2 separate charts with equivalent scales: one with the main curve and the second with a linear trendline connecting only the two RIs closest to the target range. Equations with scatter intensity as one of the variables will be specific to the instrument and settings used for analysis, and will need to be scaled using empirical reference-particle measurements.

## Supplementary information

Supplementary Information

## Data Availability

All of the flow-cytometry data are archived at FlowRepository.org. Other data are available upon request.
