# Supplementary material for: A Novel Semiconductor-Based Flow Cytometer with Enhanced Light-Scatter Sensitivity for the Analysis of Biological Nanoparticles
Source: Sci Rep. 2019 Nov 5;9:16039. doi: 10.1038/s41598-019-52366-4 (PMC6831566; doi:10.1038/s41598-019-52366-4)
Supplement: Supplementary file 1 — Supplementary Information [file 41598_2019_52366_MOESM1_ESM.pdf]

Supplementary Information

A Novel Semiconductor-Based Flow Cytometer with Enhanced Light-Scatter Sensitivity for the Analysis of Biological Nanoparticles

George C. Brittain, IV[1], Yong Q. Chen[1], Edgar Martinez[2], Vera A. Tang[3,4], Tyler M. Renner[4], Marc-André Langlois[3,4,5], and Sergei Gulnik[1]

[1]Beckman Coulter Life Sciences, Life Science Research. [2]Beckman Coulter Life Sciences, Particle Characterization. [3]University of Ottawa Flow Cytometry and Virometry Core Facility. [4]Department of Biochemistry, Microbiology and Immunology, Faculty of Medicine, University of Ottawa. [5]uOttawa Center for Infection, Immunity and Inflammation (CI3).

Running Title: **Enhanced Light-Scatter Sensitivity on the CytoFLEX**

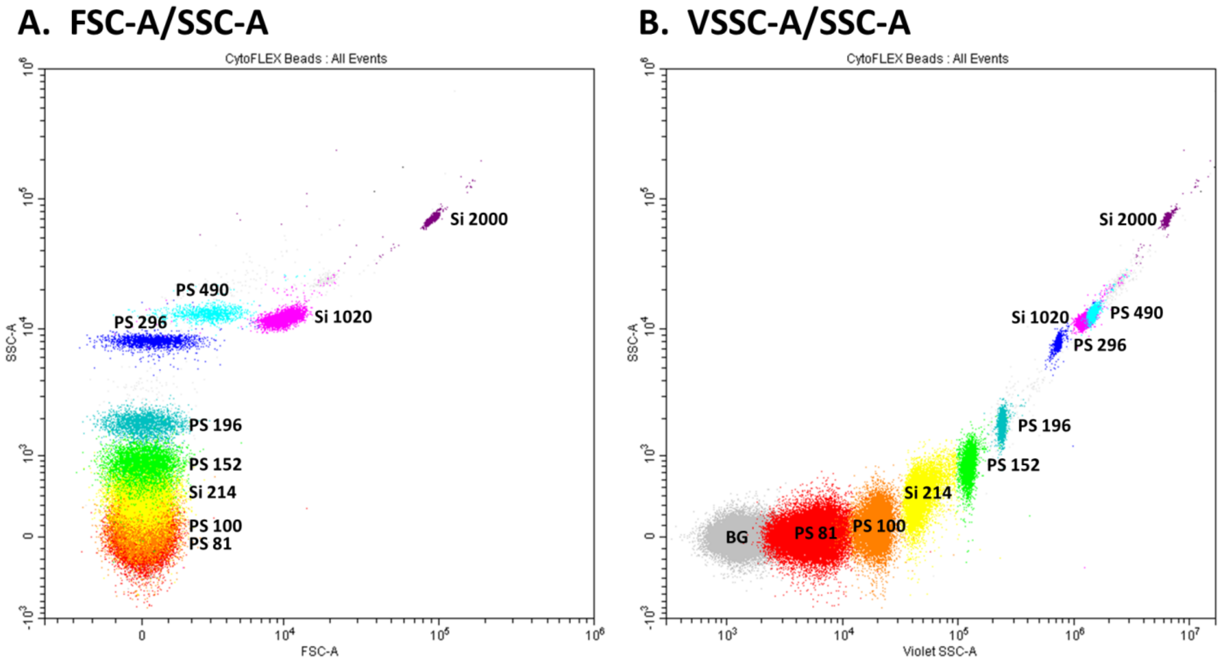

Supplementary Figure 1. FSC, SSC, and VSSC Detection on the CytoFLEX. A) FSC on the CytoFLEX uses a digital signal-analysis approach, called axial light loss detection, which can be used to resolve particles as small as 500nm, mostly independent of the refractive index or membrane integrity. B) SSC on the CytoFLEX can resolve approximately 125nm PS and 200nm Si particles, while the VSSC signal is unfiltered and can fully resolve down to at least 81nm PS. VSSC gain = 200; VSSC-H threshold = 1500.

Reference	34	35	36	37	38	Average	SD	CV (%)
Length (nm)	300	350	309	320	333	322.4	19.76	6.128
Width (nm)	230	250	237	250	268	247.0	14.56	5.895
Height (nm)	180	150	137	195	185	169.4	24.68	14.571
Ellipsoidal Volume (nm^3)	6,503,091	6,872,228	5,253,221	8,168,134	8,644,682	7,088,271	1,355,087	19.117
Spherical Diameter (nm)	231.58	235.89	215.68	249.87	254.63	**237.53**	15.49	6.523

Supplementary Table 1. Calculating an Approximate Spherical Diameter for Vaccinia. Ellipsoidal volumes were first determined based on dimensions from literature, and then the diameters of spheres of equivalent volume were calculated. The average spherical diameter was used in order to enable characterization by Mie theory.

Population Statistics

Particle	Size (nm)	VSSC-H	SD	CV (%)
PS 60nm	60	4224.7	1.84	0.043
PS 70nm	70	7512.2	27.31	0.364
PS 81nm	81	15248.5	36.07	0.237
PS 92nm	92	29488.8	132.93	0.451
PS 100nm	100	52564.6	143.39	0.273
PS 125nm	125	152420	582.43	0.382
PS 152nm	152	347417	1143.93	0.329
PS 203nm	203	924374	3790.73	0.410
PS 296nm	296	2163788	13337.5	0.616
Si 98.6nm	98.6	6401.2	37.20	0.581
Si 110nm	110	10034.8	41.01	0.409
Si 160nm	160	44581.7	217.60	0.488
Si 214nm	214	133302	55.25	0.041
Si 293nm	293	273710	237.71	0.087
HAdV-5	~95	8593.6	52.73	0.614
MLV	~110	16760.1	23.45	0.140
HSV-1	~157	79303.9	151.01	0.190
Vaccinia	~237.5	245304	1191.13	0.486
HIV-1 Peak	~100	13064.7	10.15	0.078
HIV-1 Mean	Variable	20645.9	254.65	1.233

Supplementary Table 2. The Average Population Statistics for the Data in Figure 2. All samples were read in triplicate. VSSC gain = 400; VSSC-H threshold = 3000. The VSSC-H threshold for HSV-1 and Vaccinia was 40K and 100K, respectively.

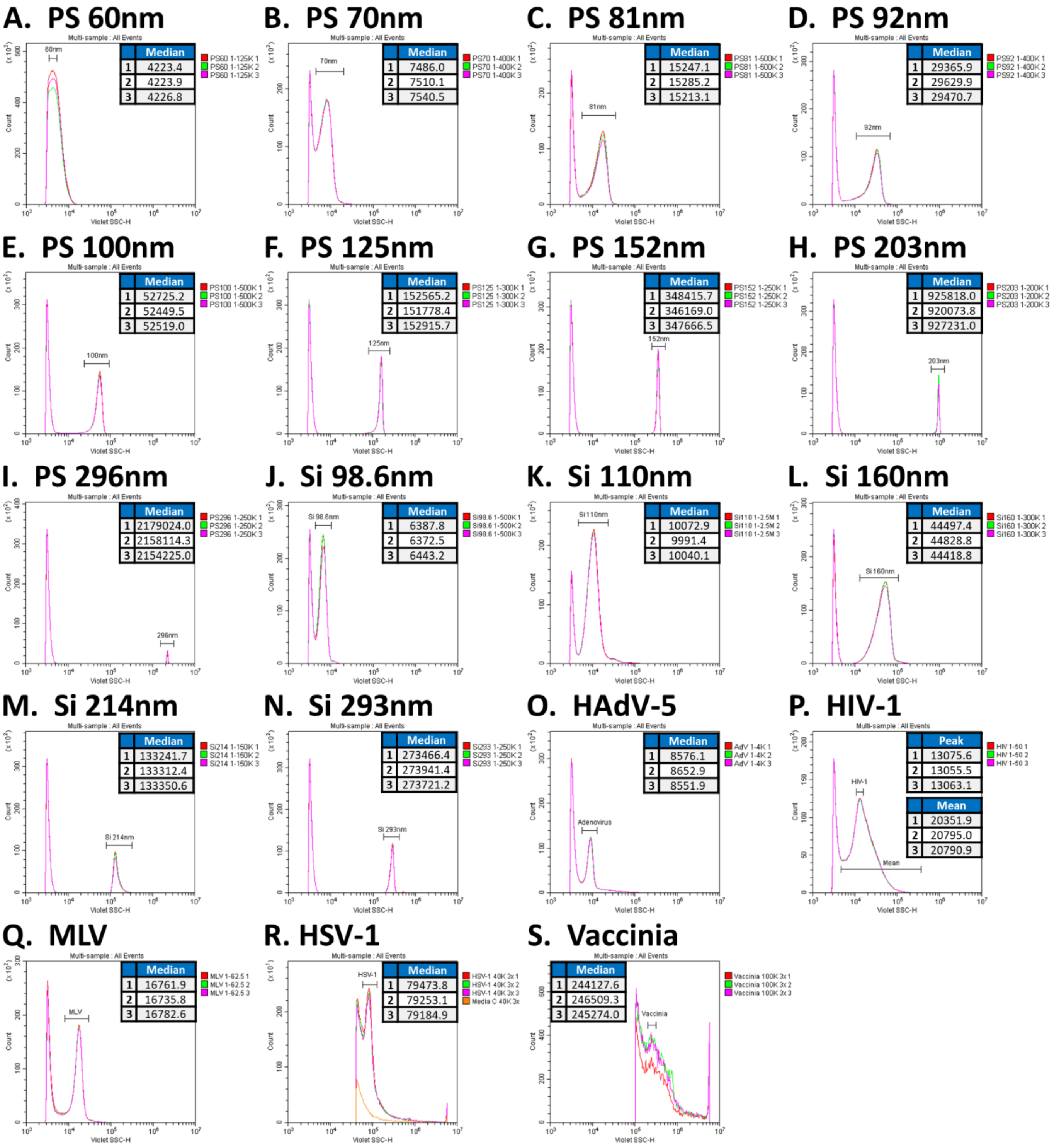

Supplementary Figure 2. Individual Measurements for Each Particle Analyzed in Figure 2. A-I) NIST-traceable PS particles between 60-296nm. J-M) Si particles between 98.6-293nm. The 98.6nm Si particles in J are NIST traceable. O-S) Viruses: HAdV-5, HIV-1, MLV, HSV-1 and Vaccinia. All samples were serially diluted and acquired in triplicate within their optimal concentration ranges. VSSC gain = 400; VSSC-H threshold = 3000. The VSSC-H threshold for HSV-1 and Vaccinia was 40K and 100K, respectively, in order to threshold out cellular debris from the freeze/thaw cell-fracture method used for preparation.

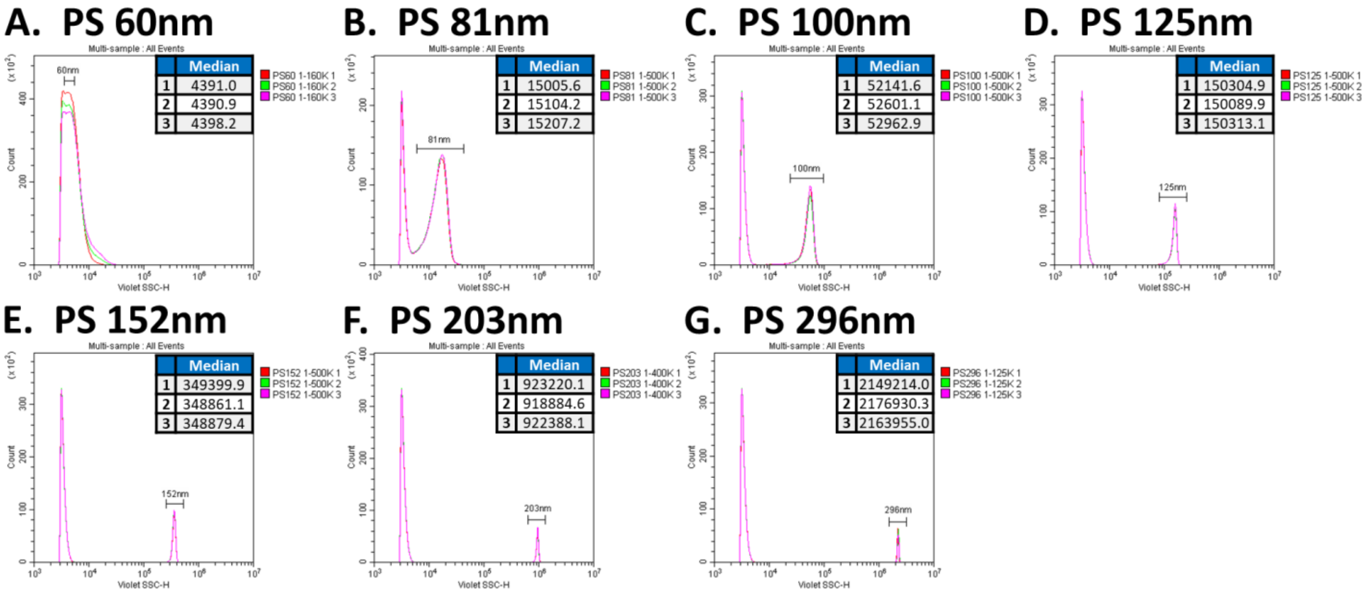

Supplementary Figure 3. Individual Measurements for Each Reference Standard Used for Scaling in Supplementary Figure 4. A-G) NIST-traceable PS particles between 60-296nm. All samples were serially diluted and acquired in triplicate within their optimal concentration ranges. VSSC gain = 400; VSSC-H threshold = 3000.

A. PS NIST Reference Standards

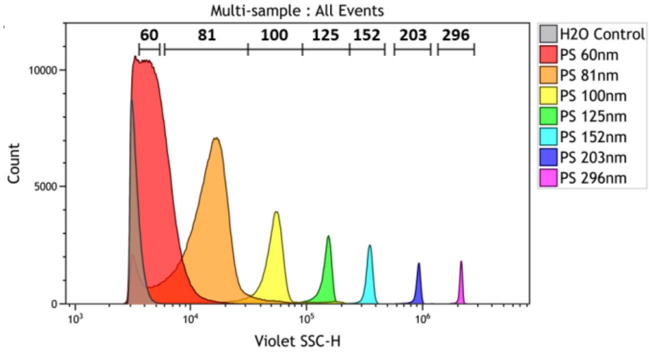

	PS 60	PS 81	PS 100	PS 125	PS 152	PS 203	PS 296
**VSSC-H**	4393.4	15105.7	52568.5	150236	349047	921498	2163366
**SD**	4.19	100.81	411.62	126.56	305.93	2300.84	13867.5
**CV (%)**	0.095	0.667	0.783	0.084	0.088	0.250	0.641
**Scatter Efficiency**	0.00699	0.02147	0.04495	0.09075	0.15388	0.32511	0.79578

B. Mie-Theory Scatter Efficiencies

RI	60nm	81nm	100nm	125nm	152nm	203nm	296nm
**1.35**	1.07E-05	3.16E-05	6.42E-05	0.00013	0.00022	0.00044	0.00108
**1.40**	0.00032	0.00095	0.00194	0.00387	0.00661	0.01332	0.03361
**1.45**	0.00106	0.00316	0.00649	0.01299	0.02214	0.04481	0.11423
**1.50**	0.00221	0.00667	0.01379	0.02767	0.04711	0.09603	0.24489
**1.55**	0.00378	0.01149	0.02388	0.04805	0.08168	0.16834	0.42515
**1.60**	0.00576	0.01762	0.03679	0.07421	0.12594	0.26349	0.65328
**1.65**	0.00812	0.02504	0.05255	0.10618	0.17992	0.38362	0.92767
**1.70**	0.01087	0.03373	0.07114	0.14393	0.24367	0.53100	1.24710
**1.75**	0.01397	0.04368	0.09253	0.18741	0.31728	0.70740	1.60800
**1.80**	0.01742	0.05484	0.11669	0.23652	0.40104	0.91307	2.00040

C. Conversion to VSSC Intensities

RI	60nm	81nm	100nm	125nm	152nm	203nm	296nm
**1.35**	6.75	22.26	75.11	210.94	493.06	1239.49	2948.54
**1.40**	201.45	669.47	2272.95	6410.99	14986.5	37745.9	91367.7
**1.45**	663.98	2223.67	7593.43	21499.6	50224.8	127019	310540
**1.50**	1391.46	4695.16	16122.9	45800.4	106860	272192	665745
**1.55**	2378.98	8087.27	27923.3	79540.6	185280	477146	1155791
**1.60**	3620.30	12398.7	43030.0	122849	285670	746841	1775973
**1.65**	5107.52	17619.2	61456.8	175778	408113	1087339	2521916
**1.70**	6832.20	23736.9	83194.6	238272	552718	1505076	3390302
**1.75**	8783.72	30733.5	108214	310252	719688	2005067	4371426
**1.80**	10951.5	38586.4	136471	391553	909681	2588022	5438184

Supplementary Figure 4. Scaling Mie-Theory Scatter-Efficiency Curves to the Real VSSC Intensities of the CytoFLEX. A) Acquisition of reference standards to scale the Mie-theory scatter-efficiency curves to the real instrument scatter intensities. 60-296nm PS particles were collected in triplicate and the population statistics are displayed in the table. B) A matrix of Mie-theory scatter efficiencies was calculated for the different-sized particles at different RIs. C) A matrix of estimated VSSC intensities, used to prepare the RI contours for a VSSC vs. Size plot, was converted from the reference particle data in A using the Mie-theory scatter efficiencies in B. All samples were collected in triplicate and these data represent the population means. VSSC gain = 400; VSSC-H threshold = 3000.

A. Approximate VSSC Intensity for 95, 100 and 110nm at RI 1.627

	PS 81	PS 100	PS 125
Average VSSC-H	15105.7	52568.5	150236
SD	100.8	411.6	126.6
CV (%)	0.67	0.78	0.08

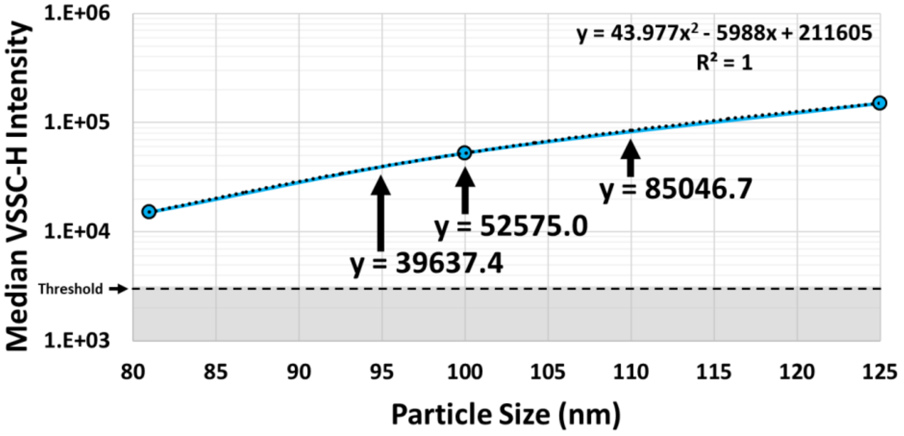

B. RI for 95nm HAdV-5

	Scatter Efficiency	Estimated VSSC
PS 95.0	0.03775	39637.4

RI	Scatter Efficiency	Conversion to VSSC
1.450	0.00548	5749.44
1.455	0.00599	6284.26
1.460	0.00652	6843.24
1.465	0.00707	7426.47
1.470	0.00765	8034.05
1.475	0.00825	8665.99
1.480	0.00888	9322.30
1.485	0.00953	10003.1
1.490	0.01020	10708.1

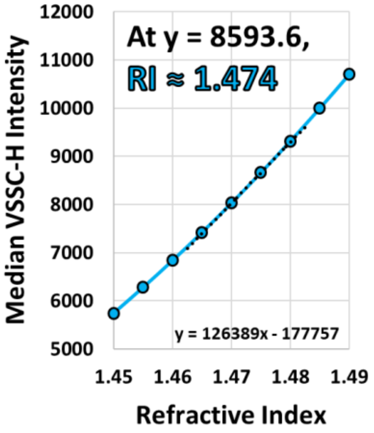

C. RI for 100nm HIV-1

	Scatter Efficiency	Estimated VSSC
PS 100.0	0.04495	52575.0

RI	Scatter Efficiency	Conversion to VSSC
1.46	0.00773	9041.46
1.465	0.00839	9813.32
1.47	0.00908	10617.5
1.475	0.00979	11454.1
1.48	0.01054	12323.5
1.485	0.01131	13224.2
1.49	0.01211	14158.7
1.495	0.01293	15126.0
1.5	0.01379	16124.9

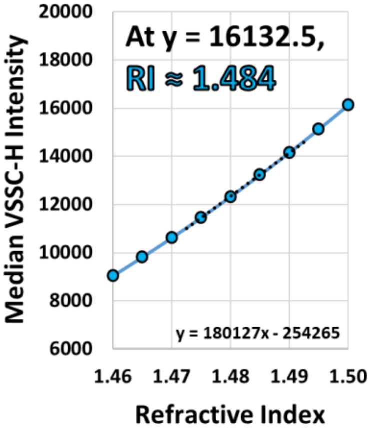

D. RI for 110nm MLV

	Scatter Efficiency	Estimated VSSC
PS 110.0	0.06143	85046.7

RI	Scatter Efficiency	Conversion to VSSC
1.450	0.00882	12210.2
1.455	0.00964	13350.1
1.460	0.01050	14542.3
1.465	0.01140	15786.9
1.470	0.01234	17084.1
1.475	0.01332	18433.9
1.480	0.01433	19836.4
1.485	0.01538	21291.4
1.490	0.01647	22800.5

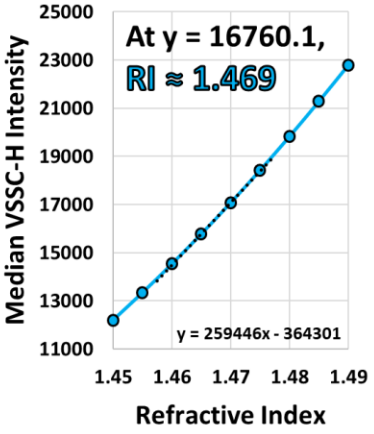

Supplementary Figure 5. Calculation of the RIs for HAdV-5, HIV-1 and MLV. A) Approximation of the VSSC intensities for PS particles at sizes equivalent to the viruses: 95, 100 and 110nm. An equation was fit to the VSSC intensity curve for the PS reference standards, and then solved for the approximate VSSC intensities at the sizes of interest. B) Calculation of the RI for HAdV-5. A matrix of Mie-theory scatter efficiencies was prepared within the range of interest, around 1.47 based on the RI contour curves from Figure 4. The scatter efficiencies were then scaled to the VSSC-intensity measurements from the CytoFLEX using the values calculated from the reference standards. The RI was calculated by finding the equation for the linear trendline connecting the 2 RI points closest to the mean VSSC intensity for the virus, and then solving for the RI at y = the measured VSSC intensity. C) Calculation of the RI for HIV-1. D) Calculation of the RI for MLV.

A. Approximate VSSC Intensity for 157 and 237.5nm at RI 1.627

	PS 125	PS 152	PS 203	PS 296
Average VSSC-H	150236	349047	921498	2163366
SD	126.6	305.9	2300.8	13867.5
CV (%)	0.08	0.09	0.25	0.64

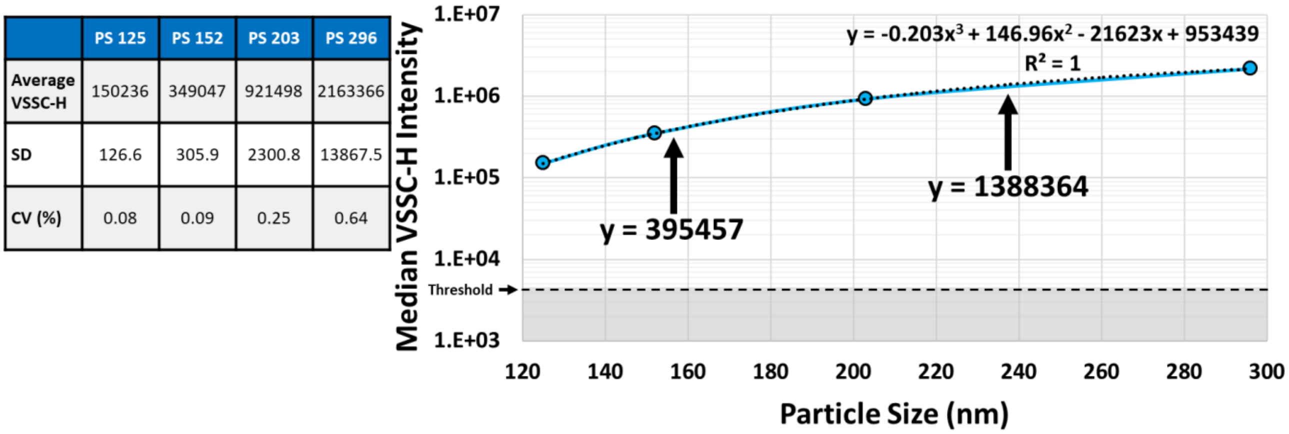

B. RI for 157nm HSV-1

	Scatter Efficiency	Estimated VSSC
PS 157.0	0.16692	395457

RI	Scatter Efficiency	Conversion to VSSC
1.450	0.02403	56932.8
1.455	0.02628	62249.1
1.460	0.02862	67807.1
1.465	0.03107	73611.5
1.470	0.03362	79657.6
1.475	0.03628	85950.0
1.480	0.03904	92486.5
1.485	0.04190	99269.3
1.490	0.04487	106296

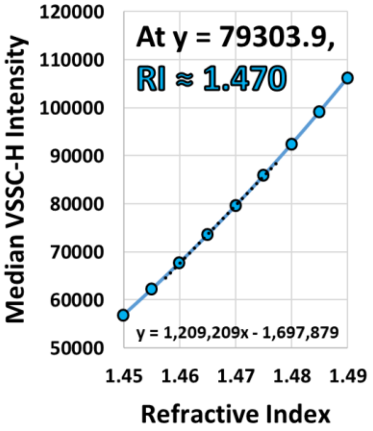

C. RI for 237.5nm Vaccinia (Spherical)

	Scatter Efficiency	Estimated VSSC
PS 237.5	0.49456	1388364

RI	Scatter Efficiency	Conversion to VSSC
1.450	0.06659	186939
1.455	0.07294	204771
1.460	0.07961	223476
1.465	0.08658	243059
1.470	0.09387	263530
1.475	0.10148	284882
1.480	0.10941	307143
1.485	0.11767	330332
1.490	0.12625	354418

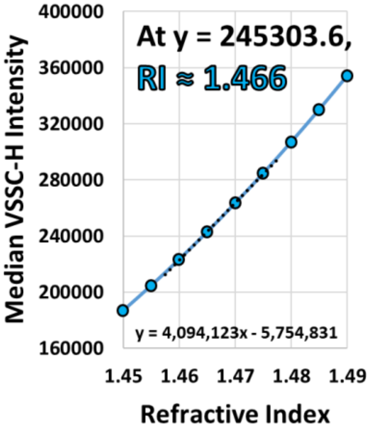

Supplementary Figure 6. Calculation of the RIs for HSV-1 and Vaccinia. A) Approximation of the VSSC intensities for PS particles at sizes equivalent to the viruses: 157 and 237.5nm. An equation was fit to the VSSC intensity curve for the PS reference standards, and then solved for the approximate VSSC intensities at the sizes of interest. B) Calculation of the RI for HSV-1. A matrix of Mie-theory scatter efficiencies was prepared within the range of interest, around 1.47. The RI was calculated by finding the equation for the trendline connecting the 2 RI points closest to the mean VSSC intensity and then solving for the RI at y = the measured VSSC intensity. C) Calculation of the RI for Vaccinia using the spherical diameter estimated in Supplementary Table 1.

A. Conversion of PS Data to VSSC Intensities at RI 1.47

Size (nm)	Scatter Efficiency at RI 1.627	Actual VSSC Intensities
**60**	0.00699	4393.37
**81**	0.02147	15105.7
**100**	0.04495	52568.5
**125**	0.09075	150236
**152**	0.15388	349047
**203**	0.32511	921498
**296**	0.79578	2163366

Size (nm)	Scatter Efficiency at RI 1.47	Conversion to VSSC at RI 1.47
**60**	0.00147	923.45
**81**	0.00441	3101.84
**100**	0.00908	10616.2
**125**	0.01818	30099.8
**152**	0.03099	70283.4
**203**	0.06287	178186
**296**	0.16047	436245

B. VSSC-H Detection Limit at RI 1.47

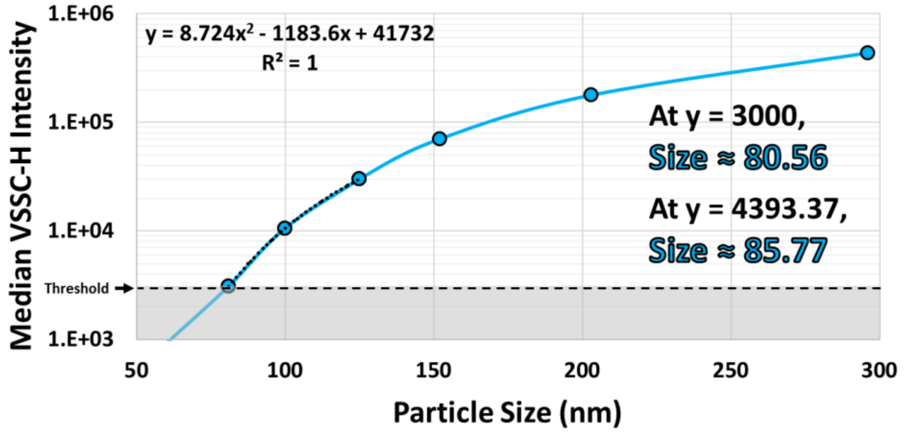

Supplementary Figure 7. Calculation of the Detection Limit for Particles with a RI of 1.47. A) Scaling a Mie-theory RI curve for particles with a RI of 1.47 to theoretical VSSC intensities using the PS reference data. B) Fitting an equation to the size range of interest and solving for the size at y = the VSSC threshold or the VSSC intensity for 60nm PS particles, which is roughly the lower VSSC detection limit, just above optical noise.

A. VSSC-H Measurements for Donor 1 CD61$^+$ Plasma EVs

Fraction 5

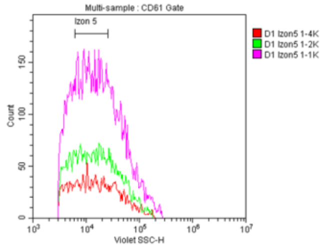

Dilution	Median		Statistics
1-4K	12348.3	Average	12388.7
1-2K	12503.2	SD	100.54
1-1K	12314.7	CV (%)	0.81

Fraction 6

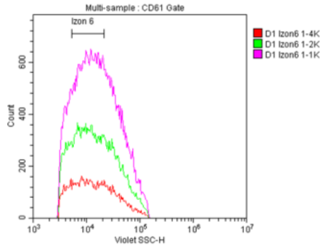

Dilution	Median		Statistics
1-4K	10431.7	Average	10578.9
1-2K	10459.3	SD	231.41
1-1K	10845.6	CV (%)	2.19

Fraction 7

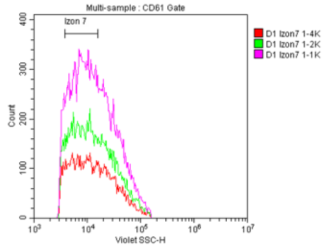

Dilution	Median		Statistics
1-4K	7617.5	Average	7707.1
1-2K	7648.1	SD	129.66
1-1K	7855.8	CV (%)	1.68

Fraction 8

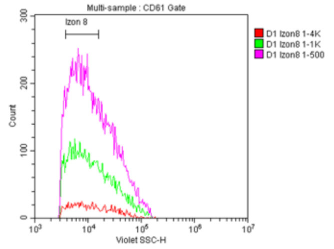

Dilution	Median		Statistics
1-4K	7465.0	Average	7439.5
1-1K	7392.6	SD	40.69
1-500	7461.0	CV (%)	0.55

B. VSSC-H Measurements for Donor 2 CD61$^+$ Plasma EVs

Fraction 5

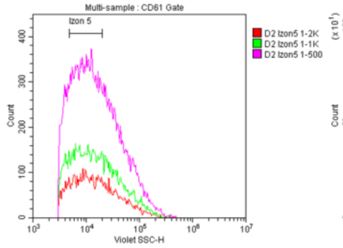

Dilution	Median		Statistics
1-2K	9720.7	Average	9707.6
1-1K	9712.0	SD	15.71
1-500	9690.2	CV (%)	0.16

Fraction 6

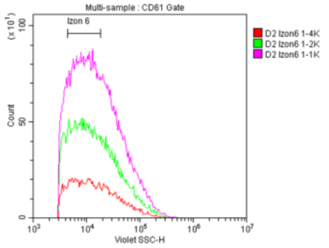

Dilution	Median		Statistics
1-4K	8670.0	Average	8738.5
1-2K	8678.5	SD	111.37
1-1K	8867.0	CV (%)	1.27

Fraction 7

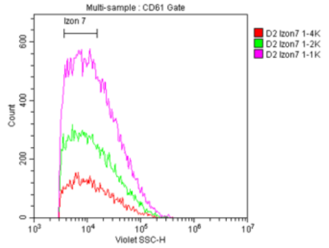

Dilution	Median		Statistics
1-4K	7561.8	Average	7532.8
1-2K	7516.0	SD	25.24
1-1K	7520.5	CV (%)	0.34

Fraction 8

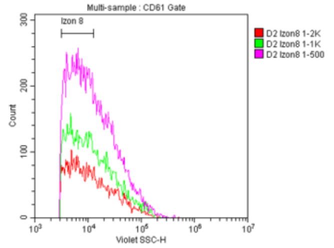

Dilution	Median		Statistics
1-2K	6100.3	Average	6173.0
1-1K	6146.5	SD	88.96
1-500	6272.2	CV (%)	1.44

C. VSSC-H Measurements for Donor 3 CD61$^+$ Plasma EVs

Fraction 5

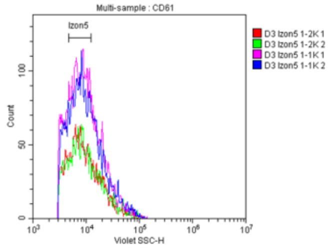

Dilution	Median		Statistics
1-2K 1	7447.5	Average	7479.5
1-2K 2	7405.6	SD	103.64
1-1K 1	7632.8	CV (%)	1.39
1-1K 2	7432.2		

Fraction 6

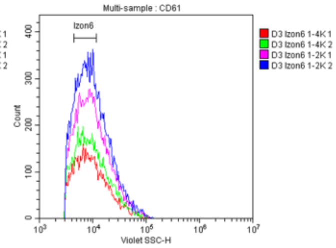

Dilution	Median		Statistics
1-4K 1	7212.5	Average	7380.8
1-4K 2	7428.3	SD	114.16
1-2K 1	7465.8	CV (%)	1.55
1-2K 2	7416.7		

Fraction 7

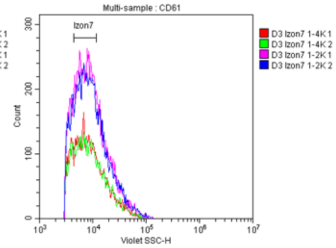

Dilution	Median		Statistics
1-4K 1	6379.9	Average	6734.3
1-4K 2	6493.0	SD	380.26
1-2K 1	7222.6	CV (%)	5.65
1-2K 2	6841.7		

Fraction 8

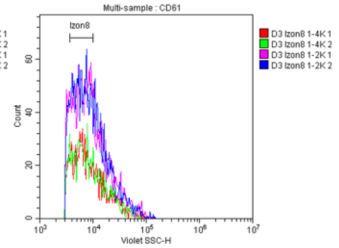

Dilution	Median		Statistics
1-4K 1	5456.5	Average	5764.8
1-4K 2	5175.6	SD	561.97
1-2K 1	6440.0	CV (%)	9.75
1-2K 2	5986.9		

D. VSSC-H Measurements for Donor 4 CD61$^+$ Plasma EVs

Fraction 5

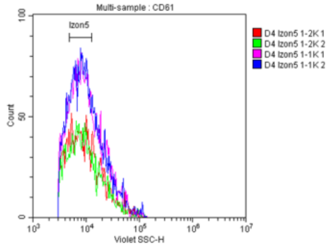

Dilution	Median		Statistics
1-2K 1	6943.8	Average	7549.2
1-2K 2	7749.5	SD	404.14
1-1K 1	7726.7	CV (%)	5.35
1-1K 2	7776.9		

Fraction 6

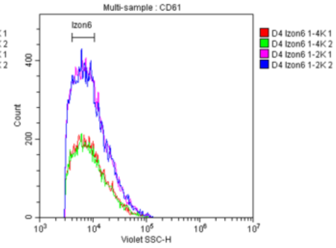

Dilution	Median		Statistics
1-4K 1	6444.4	Average	6415.0
1-4K 2	6444.8	SD	64.88
1-2K 1	6452.8	CV (%)	1.01
1-2K 2	6317.8		

Fraction 7

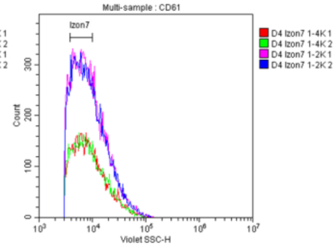

Dilution	Median		Statistics
1-4K 1	5900.0	Average	6144.1
1-4K 2	6226.9	SD	211.50
1-2K 1	6390.2	CV (%)	3.44
1-2K 2	6059.3		

Fraction 8

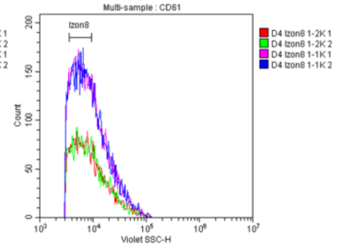

Dilution	Median		Statistics
1-2K 1	5362.2	Average	5597.9
1-2K 2	5843.9	SD	244.22
1-1K 1	5415.5	CV (%)	4.36
1-1K 2	5769.9		

Supplementary Figure 8. Serial Dilutions and Alignments of the CD61$^+$ Plasma-EV Samples. VSSC intensity measurements for A) Donor 1, B) Donor 2, C) Donor 3, and D) Donor 4 EV fractions 5-8. The median gates were located at the central population distribution in order to eliminate bias due to a portion of the lower population distributions being cutoff. The population statistics for each sample can be found in the tables. VSSC gain = 400; VSSC-H threshold = 3000.

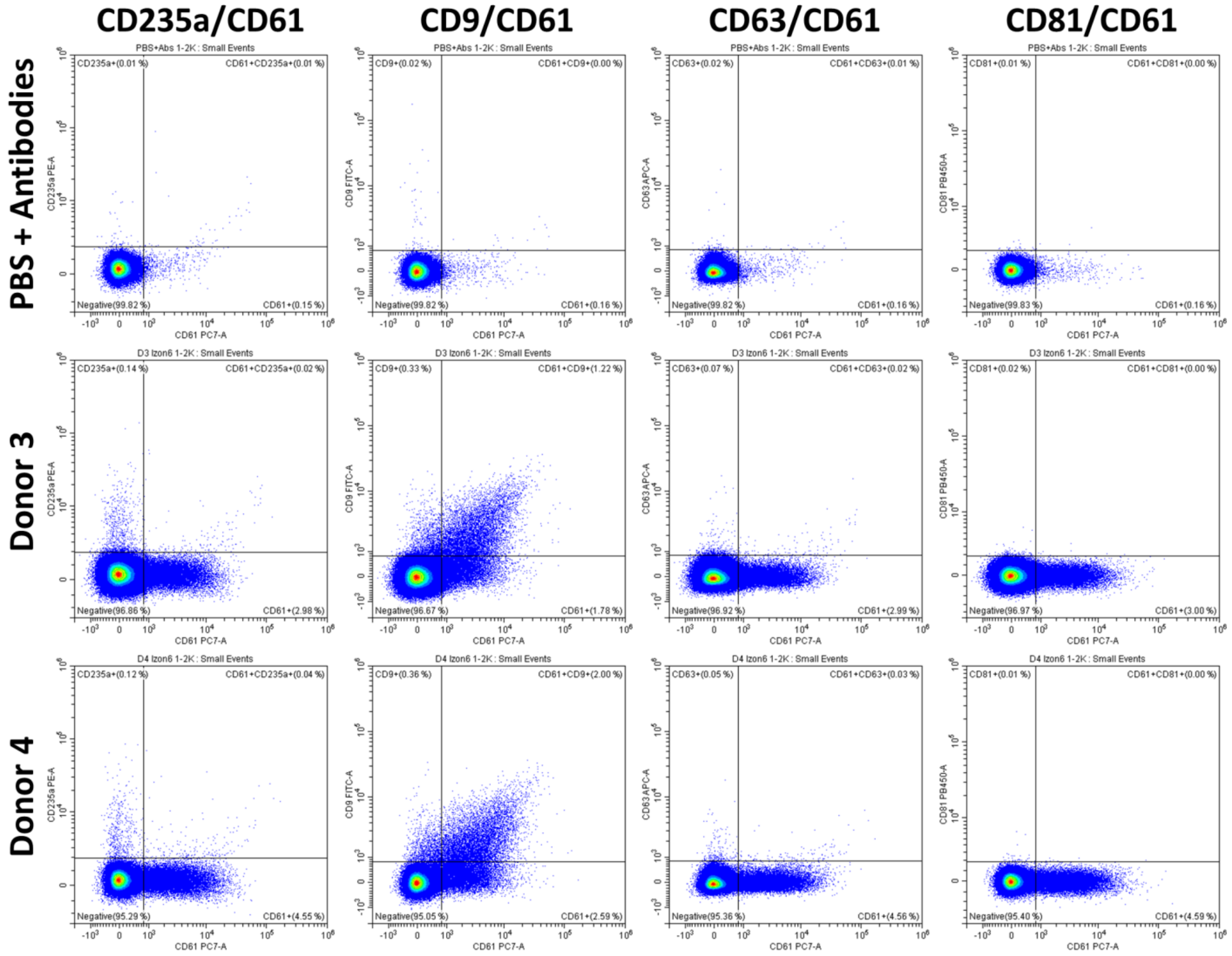

Supplementary Figure 9. Tetraspanin Expression on the CD61+ Plasma EVs from Donors 3 and 4. The CD61+ EVs were 40.7% and 43.6% CD9+ for Donor 3 and 4, respectively. CD63 and CD81 expression was absent, and they were CD235a negative. The PBS + antibody control shows minimal antibody aggregates. These samples were serially diluted and acquired in quadruplicate. VSSC gain = 400; VSSC-H threshold = 3000.

A. Median Diameter - Donor 1

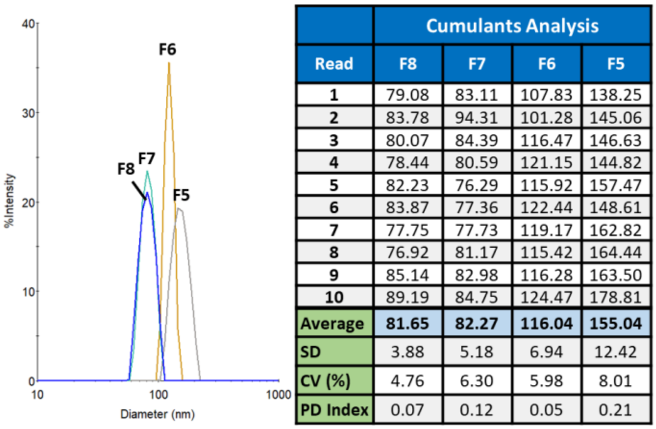

	Cumulants Analysis			
Read	F8	F7	F6	F5
1	79.08	83.11	107.83	138.25
2	83.78	94.31	101.28	145.06
3	80.07	84.39	116.47	146.63
4	78.44	80.59	121.15	144.82
5	82.23	76.29	115.92	157.47
6	83.87	77.36	122.44	148.61
7	77.75	77.73	119.17	162.82
8	76.92	81.17	115.42	164.44
9	85.14	82.98	116.28	163.50
10	89.19	84.75	124.47	178.81
**Average**	**81.65**	**82.27**	**116.04**	**155.04**
**SD**	3.88	5.18	6.94	12.42
**CV (%)**	4.76	6.30	5.98	8.01
**PD Index**	0.07	0.12	0.05	0.21

B. Median Diameter - Donor 2

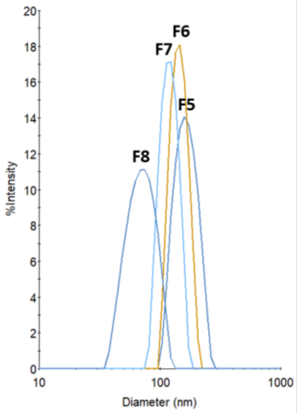

	Cumulants Analysis			
Read	F8	F7	F6	F5
1	72.37	95.28	130.12	148.61
2	64.03	90.70	118.77	144.18
3	67.99	91.38	116.08	149.22
4	66.37	100.44	122.82	153.82
5	74.42	110.27	131.86	138.39
6	81.15	114.76	137.62	158.30
7	79.76	116.92	137.60	168.67
8	78.50	119.24	138.56	140.16
9	69.49	117.81	135.17	166.81
10	62.49	113.15	133.08	170.03
**Average**	**71.66**	**106.99**	**130.17**	**153.82**
**SD**	6.66	11.37	8.16	11.73
**CV (%)**	9.29	10.63	6.27	7.62
**PD Index**	0.17	0.02	0.01	0.34

C. Median Diameter - Donor 3

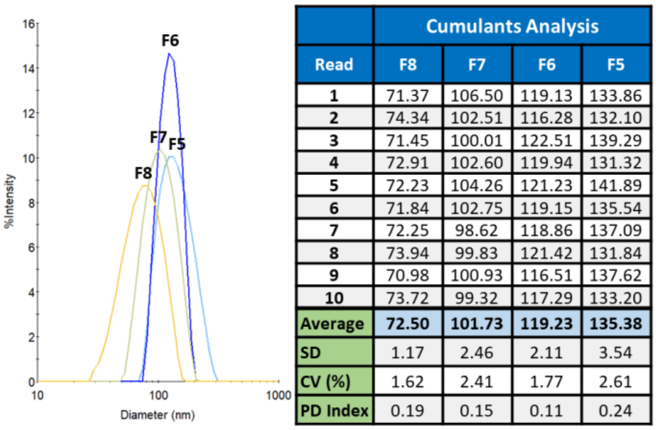

	Cumulants Analysis			
Read	F8	F7	F6	F5
1	71.37	106.50	119.13	133.86
2	74.34	102.51	116.28	132.10
3	71.45	100.01	122.51	139.29
4	72.91	102.60	119.94	131.32
5	72.23	104.26	121.23	141.89
6	71.84	102.75	119.15	135.54
7	72.25	98.62	118.86	137.09
8	73.94	99.83	121.42	131.84
9	70.98	100.93	116.51	137.62
10	73.72	99.32	117.29	133.20
**Average**	**72.50**	**101.73**	**119.23**	**135.38**
**SD**	1.17	2.46	2.11	3.54
**CV (%)**	1.62	2.41	1.77	2.61
**PD Index**	0.19	0.15	0.11	0.24

D. Median Diameter - Donor 4

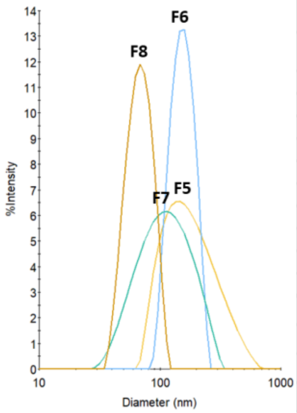

	Cumulants Analysis			
Read	F8	F7	F6	F5
1	61.49	106.47	146.17	179.42
2	61.22	108.77	140.18	184.52
3	64.25	111.23	145.81	182.07
4	66.04	115.32	153.45	179.42
5	65.39	109.48	158.94	205.38
6	67.27	110.47	155.65	211.39
7	67.82	102.00	154.55	188.81
8	67.70	104.81	153.15	198.26
9	63.15	102.79	149.88	210.73
10	63.54	107.06	141.88	203.05
**Average**	**64.78**	**107.84**	**149.96**	**194.30**
**SD**	2.45	4.07	6.23	12.89
**CV (%)**	3.78	3.78	4.15	6.63
**PD Index**	0.38	0.23	0.23	0.43

Supplementary Figure 10. DLS Measurements for the Plasma-EV Samples. DLS size measurements for A) Donor 1, B) Donor 2, C) Donor 3, and D) Donor 4 EV fractions 5-8. Each sample was read 10x for 10x 2-second acquisitions per read: 100 acquisitions in total. The population statistics for each sample can be found in the tables.

A. Approximate VSSC-H for 81.7, 82.3, 116.0, and 155.0nm at RI 1.627

	VSSC-H	SD	CV (%)
PS 60nm	4393.4	4.19	0.095
PS 81nm	15105.7	100.81	0.667
PS 100nm	52568.5	411.62	0.783
PS 125nm	150236	126.56	0.084
PS 152nm	349047	305.93	0.088
PS 203nm	921498	2300.84	0.250

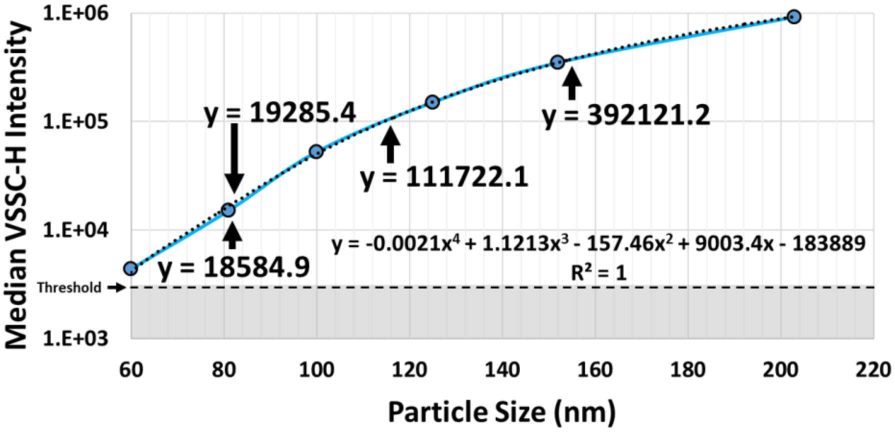

B. RI for Donor 1 – 81.7nm EVs

	Scatter Efficiency	Estimated VSSC
PS 81.7	0.02215	18584.9

RI	Scatter Efficiency	Conversion to VSSC
1.500	0.00688	5773.18
1.505	0.00732	6139.55
1.510	0.00777	6517.18
1.515	0.00823	6906.21
1.520	0.00871	7306.58
1.525	0.00920	7718.27
1.530	0.00970	8141.29
1.535	0.01022	8575.39
1.540	0.01075	9021.82

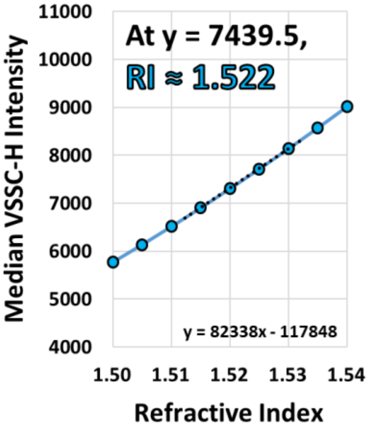

C. RI for Donor 1 – 82.3nm EVs

	Scatter Efficiency	Estimated VSSC
PS 82.3	0.02274	19285.4

RI	Scatter Efficiency	Conversion to VSSC
1.500	0.00706	5987.88
1.505	0.00751	6367.89
1.510	0.00797	6759.77
1.515	0.00845	7163.35
1.520	0.00894	7578.81
1.525	0.00944	8005.97
1.530	0.00996	8445.01
1.535	0.01049	8896.00
1.540	0.01104	9358.18

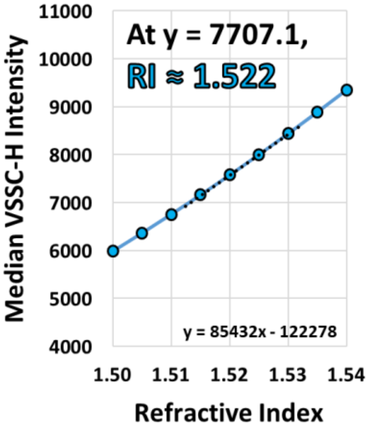

D. RI for Donor 1 – 116.0nm EVs

	Scatter Efficiency	Estimated VSSC
PS 116.0	0.07255	111722

RI	Scatter Efficiency	Conversion to VSSC
1.410	0.00421	6485.22
1.415	0.00483	7439.47
1.420	0.00549	8460.54
1.425	0.00620	9548.90
1.430	0.00695	10704.6
1.435	0.00775	11927.8
1.440	0.00859	13219.0
1.445	0.00947	14578.2
1.450	0.01039	16005.2

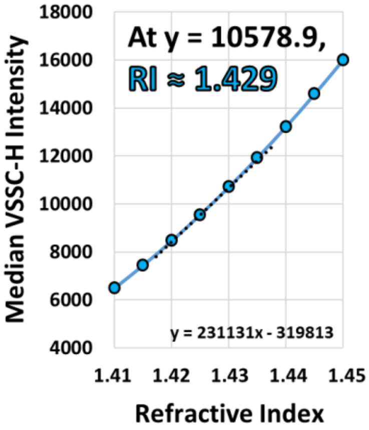

E. RI for Donor 1 – 155.0nm EVs

	Scatter Efficiency	Estimated VSSC
PS 155.0	0.16164	392121

RI	Scatter Efficiency	Conversion to VSSC
1.370	0.00179	4333.86
1.375	0.00241	5844.46
1.380	0.00313	7583.82
1.385	0.00394	9552.68
1.390	0.00484	11752.0
1.395	0.00585	14183.0
1.400	0.00694	16846.4
1.405	0.00814	19743.1
1.410	0.00943	22874.2

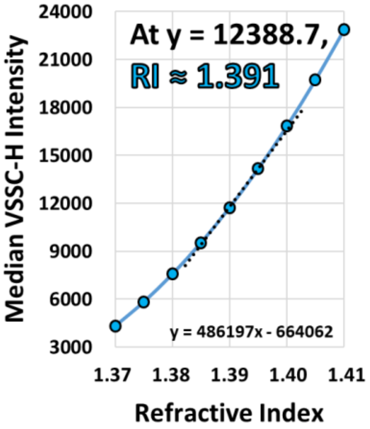

Supplementary Figure 11. Calculation of the RIs for the Donor 1 CD61$^+$ Plasma-EV Fractions. A) Approximation of the VSSC-H intensities for particles of equivalent size to the different EV fractions: 81.7, 82.3, 116.0, and 155.0nm. An equation was fit to the curve for the VSSC intensities of the PS reference standards, and was then solved for the expected VSSC intensities at the sizes of interest. B-E) Calculation of the RIs for the 81.7, 82.3, 116.0, and 155.0nm CD61$^+$ EVs from Donor 1, respectively. Similar to Supplementary Figure 5, the RI range was narrowed to a distribution +/- 0.02 around the RI estimated from Figure 4, using 0.005 increments. The calculated VSSC intensities were plotted vs. RI, and the equations were determined for the trendline connecting the 2 RI points closest to the mean VSSC intensity for each population. These equations were solved to identify the RIs at y = the mean VSSC intensity for each population of interest.

A. Approximate VSSC-H for 71.7, 107.0, 130.2, and 153.8nm at RI 1.627

	VSSC-H	SD	CV (%)
PS 60nm	4393.4	4.19	0.095
PS 81nm	15105.7	100.81	0.667
PS 100nm	52568.5	411.62	0.783
PS 125nm	150236	126.56	0.084
PS 152nm	349047	305.93	0.088
PS 203nm	921498	2300.84	0.250

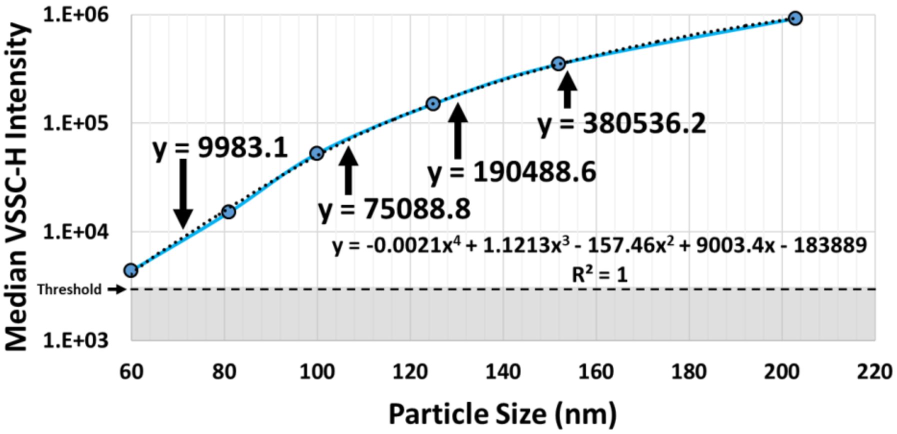

B. RI for Donor 2 – 71.7nm EVs

	Scatter Efficiency	Estimated VSSC
PS 71.7	0.01370	9983.10

RI	Scatter Efficiency	Conversion to VSSC
1.550	0.00737	5371.29
1.555	0.00773	5628.41
1.560	0.00809	5891.43
1.565	0.00846	6160.42
1.570	0.00883	6435.32
1.575	0.00922	6716.04
1.580	0.00961	7002.74
1.585	0.01001	7295.34
1.590	0.01042	7593.33

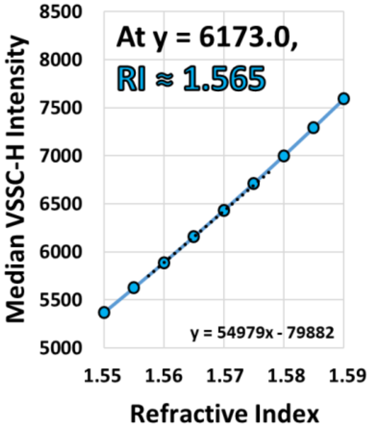

C. RI for Donor 2 – 107.0nm EVs

	Scatter Efficiency	Estimated VSSC
PS 107.0	0.05620	75088.8

RI	Scatter Efficiency	Conversion to VSSC
1.410	0.00328	4379.69
1.415	0.00376	5023.38
1.420	0.00428	5711.95
1.425	0.00482	6445.82
1.430	0.00541	7224.85
1.435	0.00603	8049.43
1.440	0.00668	8919.43
1.445	0.00736	9835.13
1.450	0.00808	10796.7

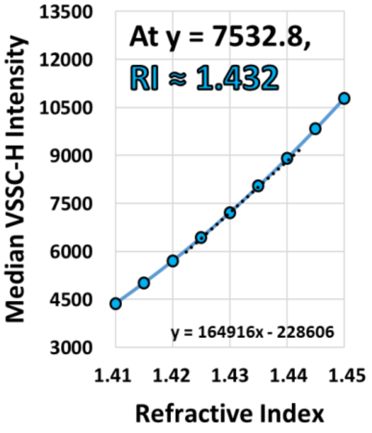

D. RI for Donor 2 – 130.2nm EVs

	Scatter Efficiency	Estimated VSSC
PS 130.2	0.10198	190489

RI	Scatter Efficiency	Conversion to VSSC
1.380	0.00196	3659.41
1.385	0.00247	4609.61
1.390	0.00304	5671.32
1.395	0.00366	6844.74
1.400	0.00435	8130.79
1.405	0.00510	9529.66
1.410	0.00591	11041.9
1.415	0.00678	12667.9
1.420	0.00771	14408.3

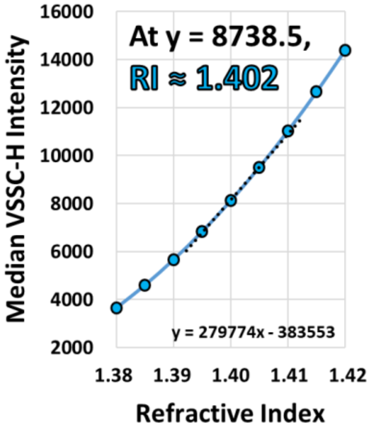

E. RI for Donor 2 – 153.8nm EVs

	Scatter Efficiency	Estimated VSSC
PS 153.8	0.15852	380536

RI	Scatter Efficiency	Conversion to VSSC
1.370	0.00175	4204.57
1.375	0.00236	5670.35
1.380	0.00307	7357.71
1.385	0.00386	9267.83
1.390	0.00475	11401.9
1.395	0.00573	13760.5
1.400	0.00681	16344.7
1.405	0.00798	19155.2
1.410	0.00925	22193.1

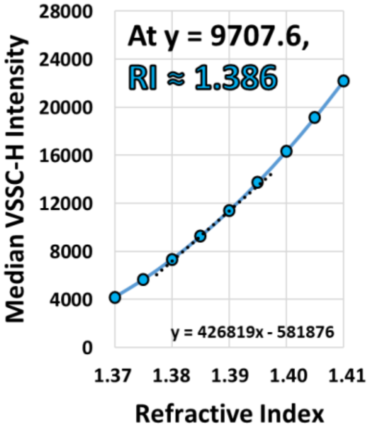

Supplementary Figure 12. Calculation of the RIs for the Donor 2 $CD61^+$ Plasma-EV Fractions. A) Approximation of the VSSC-H intensities for particles of equivalent size to the different EV fractions: 71.7, 107.0, 130.2, and 153.8nm. An equation was fit to the curve for the VSSC intensities of the PS reference standards, and was then solved for the expected VSSC intensities at the sizes of interest. B-E) Calculation of the RIs for the 71.7, 107.0, 130.2, and 153.8nm $CD61^+$ EVs from Donor 2, respectively. This was performed as described for Donor 1 in Supplementary Figure 11.

A. Approximate VSSC-H for 72.5, 101.7, 119.2, and 135.4nm at RI 1.627

	VSSC-H	SD	CV (%)
PS 60nm	4393.4	4.19	0.095
PS 81nm	15105.7	100.81	0.667
PS 100nm	52568.5	411.62	0.783
PS 125nm	150236	126.56	0.084
PS 152nm	349047	305.93	0.088
PS 203nm	921498	2300.84	0.250

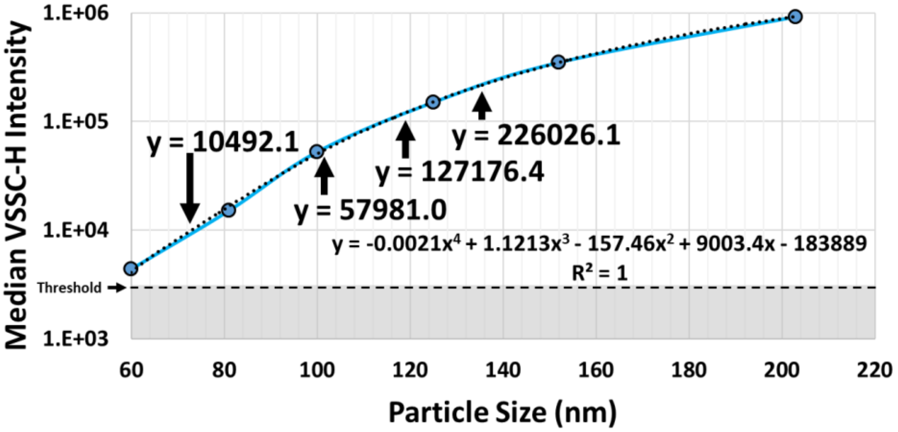

B. RI for Donor 3 – 72.5nm EVs

	Scatter Efficiency	Estimated VSSC
PS 72.5	0.01428	10492.1

RI	Scatter Efficiency	Conversion to VSSC
1.530	0.00629	4624.31
1.535	0.00663	4869.50
1.540	0.00697	5121.00
1.545	0.00732	5378.74
1.550	0.00768	5642.74
1.555	0.00805	5912.97
1.560	0.00842	6189.53
1.565	0.00881	6472.26
1.570	0.00920	6761.23

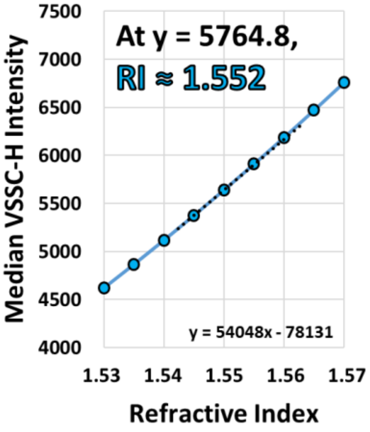

C. RI for Donor 3 – 101.7nm EVs

	Scatter Efficiency	Estimated VSSC
PS 101.7	0.04756	57981.0

RI	Scatter Efficiency	Conversion to VSSC
1.420	0.00363	4428.37
1.425	0.00410	4996.74
1.430	0.00459	5599.99
1.435	0.00512	6238.34
1.440	0.00567	6911.93
1.445	0.00625	7620.63
1.450	0.00686	8364.81
1.455	0.00750	9144.34
1.460	0.00817	9959.35

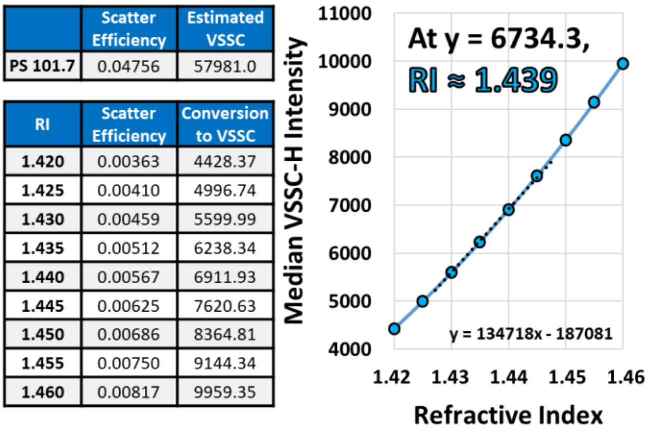

D. RI for Donor 3 – 119.2nm EVs

	Scatter Efficiency	Estimated VSSC
PS 119.2	0.07883	127176

RI	Scatter Efficiency	Conversion to VSSC
1.390	0.00235	3789.20
1.395	0.00283	4572.96
1.400	0.00337	5431.73
1.405	0.00395	6365.68
1.410	0.00457	7375.29
1.415	0.00524	8460.73
1.420	0.00596	9622.49
1.425	0.00673	10860.5
1.430	0.00755	12175.4

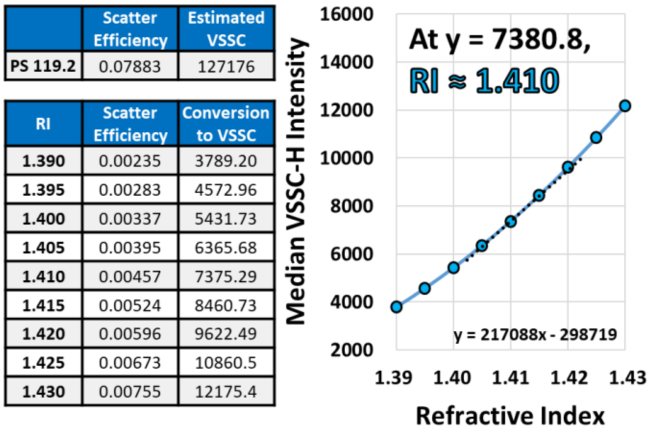

E. RI for Donor 3 – 135.4nm EVs

	Scatter Efficiency	Estimated VSSC
PS 135.4	0.11368	226026.1

RI	Scatter Efficiency	Conversion to VSSC
1.370	0.00125	2483.15
1.375	0.00168	3349.04
1.380	0.00219	4345.75
1.385	0.00275	5474.49
1.390	0.00339	6735.45
1.395	0.00409	8129.23
1.400	0.00486	9656.61
1.405	0.00569	11318.0
1.410	0.00660	13114.0

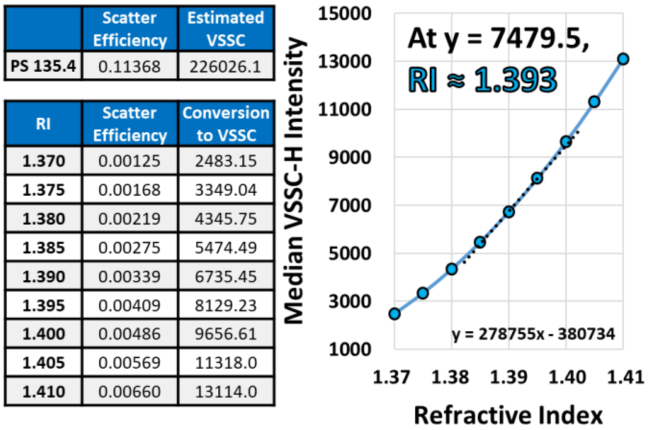

Supplementary Figure 13. Calculation of the RIs for the Donor 3 CD61$^+$ Plasma-EV Fractions. A) Approximation of the VSSC-H intensities for particles of equivalent size to the different EV fractions: 72.5, 101.7, 119.2, and 135.4nm. An equation was fit to the curve for the VSSC intensities of the PS reference standards, and was then solved for the expected VSSC intensities at the sizes of interest. B-E) Calculation of the RIs for the 72.5, 101.7, 119.2, and 135.4nm CD61$^+$ EVs from Donor 3, respectively. This was performed as described for Donor 1 in Supplementary Figure 11.

A. Approximate VSSC-H for 64.8, 107.8, 150.0, and 194.3nm at RI 1.627

	VSSC-H	SD	CV (%)
PS 60nm	4393.4	4.19	0.095
PS 81nm	15105.7	100.81	0.667
PS 100nm	52568.5	411.62	0.783
PS 125nm	150236	126.56	0.084
PS 152nm	349047	305.93	0.088
PS 203nm	921498	2300.84	0.250

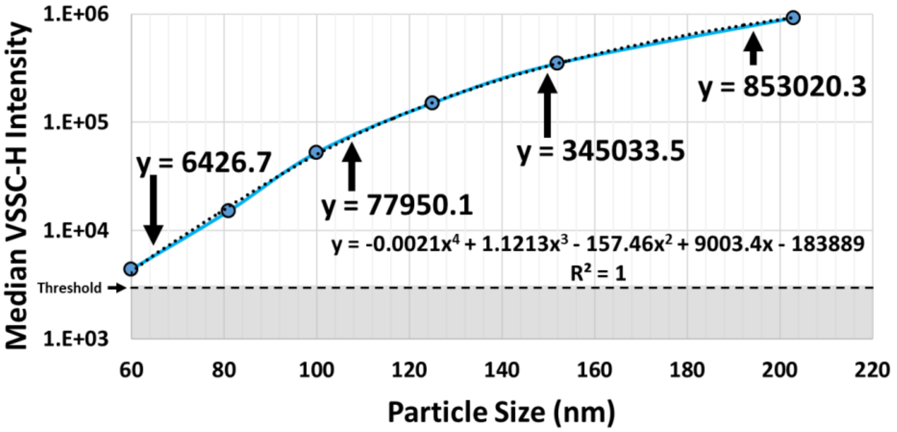

B. RI for Donor 4 – 64.8nm EVs

	Scatter Efficiency	Estimated VSSC
PS 64.8	0.00937	6426.70

RI	Scatter Efficiency	Conversion to VSSC
1.590	0.00714	4897.27
1.595	0.00742	5092.26
1.600	0.00771	5290.96
1.605	0.00801	5493.23
1.610	0.00831	5699.20
1.615	0.00861	5908.82
1.620	0.00892	6122.06
1.625	0.00924	6338.94
1.630	0.00956	6559.40

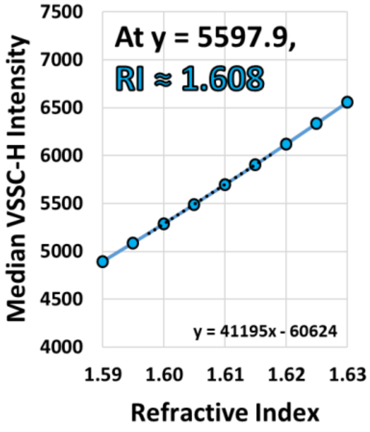

C. RI for Donor 4 – 107.8nm EVs

	Scatter Efficiency	Estimated VSSC
PS 107.8	0.05758	77950.1

RI	Scatter Efficiency	Conversion to VSSC
1.400	0.00247	3347.76
1.405	0.00290	3922.76
1.410	0.00336	4544.05
1.415	0.00385	5211.93
1.420	0.00438	5926.51
1.425	0.00494	6688.07
1.430	0.00554	7496.48
1.435	0.00617	8352.14
1.440	0.00684	9255.04

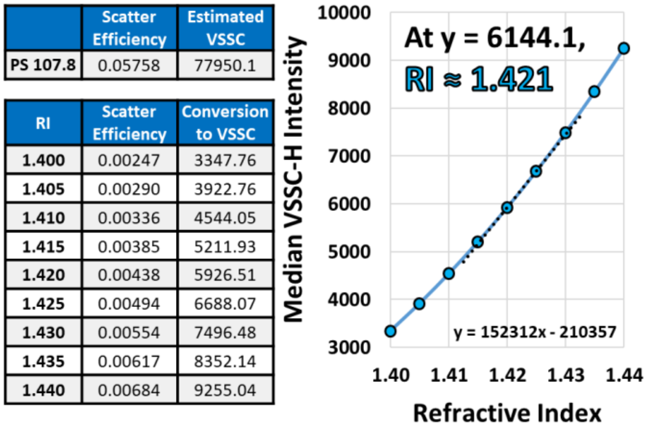

D. RI for Donor 4 – 150.0nm EVs

	Scatter Efficiency	Estimated VSSC
PS 150.0	0.14880	345034

RI	Scatter Efficiency	Conversion to VSSC
1.360	0.00076	1751.83
1.365	0.00116	2680.50
1.370	0.00164	3808.35
1.375	0.00222	5136.08
1.380	0.00287	6664.62
1.385	0.00362	8395.12
1.390	0.00445	10328.3
1.395	0.00538	12465.0
1.400	0.00639	14806.3

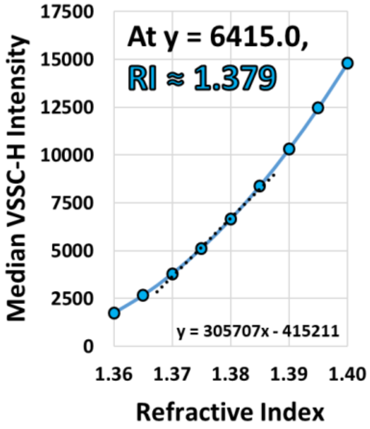

E. RI for Donor 4 – 194.3nm EVs

	Scatter Efficiency	Estimated VSSC
PS 194.3	0.28825	853020

RI	Scatter Efficiency	Conversion to VSSC
1.350	0.00039	1167.92
1.355	0.00083	2448.09
1.360	0.00142	4200.44
1.365	0.00217	6427.32
1.370	0.00309	9131.24
1.375	0.00416	12314.6
1.380	0.00540	15980.0
1.385	0.00680	20129.8
1.390	0.00837	24766.1

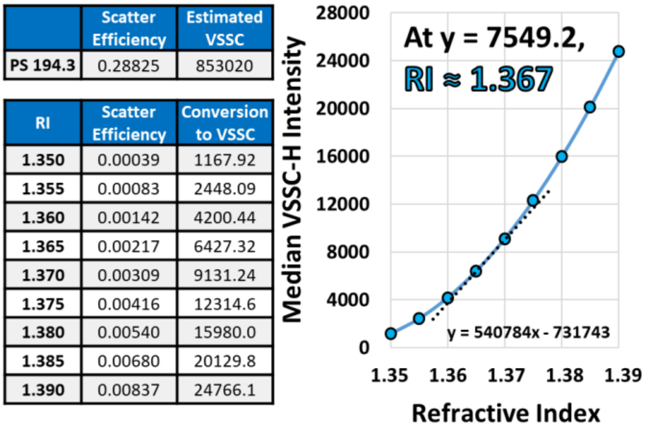

Supplementary Figure 14. Calculation of the RIs for the Donor 4 CD61$^+$ Plasma-EV Fractions. A) Approximation of the VSSC-H intensities for particles of equivalent size to the different EV fractions: 64.8, 107.8, 150.0, and 194.3nm. An equation was fit to the curve for the VSSC intensities of the PS reference standards, and was then solved for the expected VSSC intensities at the sizes of interest. B-E) Calculation of the RIs for the 64.8, 107.8, 150.0, and 194.3nm CD61$^+$ EVs from Donor 4, respectively. This was performed as described for Donor 1 in Supplementary Figure 11.

A. Si 68.6nm

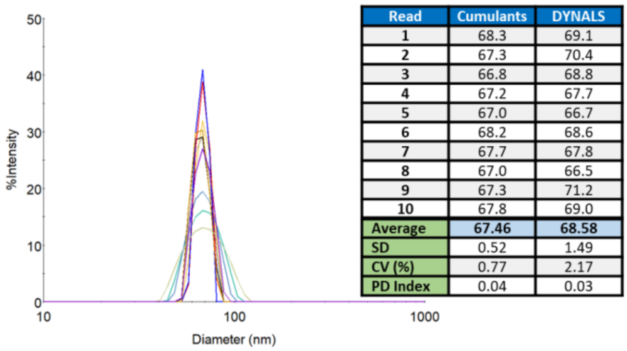

Read	Cumulants	DYNALS
1	68.3	69.1
2	67.3	70.4
3	66.8	68.8
4	67.2	67.7
5	67.0	66.7
6	68.2	68.6
7	67.7	67.8
8	67.0	66.5
9	67.3	71.2
10	67.8	69.0
Average	67.46	68.58
SD	0.52	1.49
CV (%)	0.77	2.17
PD Index	0.04	0.03

B. Si 98.6nm

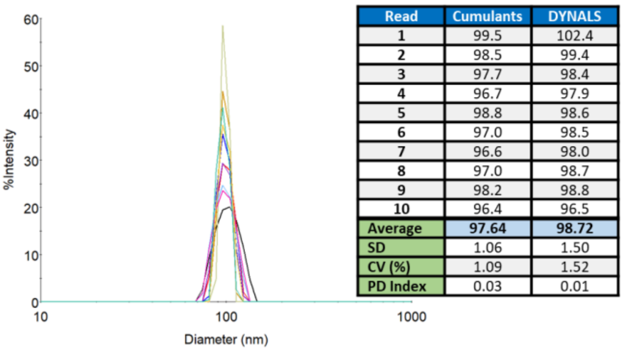

Read	Cumulants	DYNALS
1	99.5	102.4
2	98.5	99.4
3	97.7	98.4
4	96.7	97.9
5	98.8	98.6
6	97.0	98.5
7	96.6	98.0
8	97.0	98.7
9	98.2	98.8
10	96.4	96.5
Average	97.64	98.72
SD	1.06	1.50
CV (%)	1.09	1.52
PD Index	0.03	0.01

C. Si 160nm

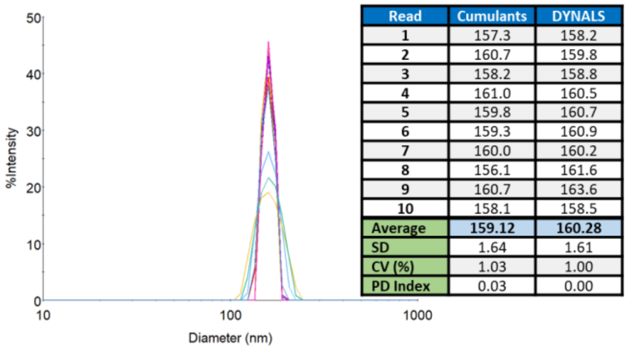

Read	Cumulants	DYNALS
1	157.3	158.2
2	160.7	159.8
3	158.2	158.8
4	161.0	160.5
5	159.8	160.7
6	159.3	160.9
7	160.0	160.2
8	156.1	161.6
9	160.7	163.6
10	158.1	158.5
Average	159.12	160.28
SD	1.64	1.61
CV (%)	1.03	1.00
PD Index	0.03	0.00

D. Si 214nm

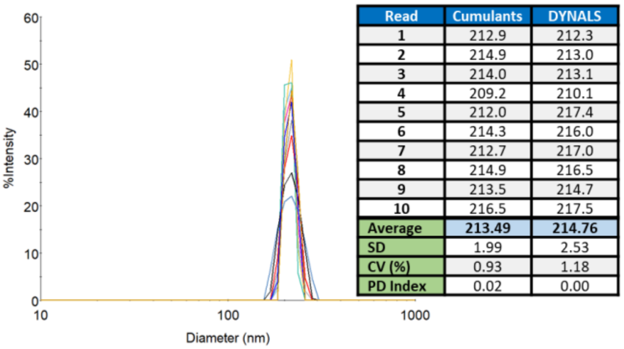

Read	Cumulants	DYNALS
1	212.9	212.3
2	214.9	213.0
3	214.0	213.1
4	209.2	210.1
5	212.0	217.4
6	214.3	216.0
7	212.7	217.0
8	214.9	216.5
9	213.5	214.7
10	216.5	217.5
Average	213.49	214.76
SD	1.99	2.53
CV (%)	0.93	1.18
PD Index	0.02	0.00

Supplementary Figure 15. Standardization of the DelsaMax Pro DLS Analyzer. A) 68.6nm Si NIST beads. B) 98.6nm Si NIST beads. C) 160nm Si beads. D) 214nm Si beads. Each sample was read 10x for 10x 2-second acquisitions per read. The mean diameters from both Cumulants and DYNALS analyses are displayed, along with the average, SD, CV and PD Index for each data set.
